# Clinical-grade human umbilical cord-derived mesenchymal stem cells improved skeletal muscle dysfunction in age-associated sarcopenia mice

**DOI:** 10.1038/s41419-023-05843-8

**Published:** 2023-05-12

**Authors:** Chao Wang, Bichun Zhao, Jinglei Zhai, Ailin Wang, Ning Cao, Tuling Liao, Ruyu Su, Lijuan He, Yanhua Li, Xuetao Pei, Yali Jia, Wen Yue

**Affiliations:** 1 Stem Cell and Regenerative Medicine Lab, Beijing Institute of Radiation Medicine, Beijing, 100850 China; 2 State Key Laboratory of Stem Cell and Reproductive Biology, Institute of Zoology, Chinese Academy of Sciences, Beijing, 100101 China; 3 920th Hospital of Joint Logistics Support Force, Kunming, 650032 China; 4 South China Institute of Biomedicine, Guangzhou, 510005 China

**Keywords:** Mesenchymal stem cells, Stem-cell research

## Abstract

With the expansion of the aging population, age-associated sarcopenia (AAS) has become a severe clinical disease of the elderly and a key challenge for healthy aging. Regrettably, no approved therapies currently exist for treating AAS. In this study, clinical-grade human umbilical cord-derived mesenchymal stem cells (hUC-MSCs) were administrated to two classic mouse models (SAMP8 mice and D-galactose-induced aging mice), and their effects on skeletal muscle mass and function were investigated by behavioral tests, immunostaining, and western blotting. Core data results showed that hUC-MSCs significantly restored skeletal muscle strength and performance in both mouse models via mechanisms including raising the expression of crucial extracellular matrix proteins, activating satellite cells, enhancing autophagy, and impeding cellular aging. For the first time, the study comprehensively evaluates and demonstrates the preclinical efficacy of clinical-grade hUC-MSCs for AAS in two mouse models, which not only provides a novel model for AAS, but also highlights a promising strategy to improve and treat AAS and other age-associated muscle diseases.

**Figure:**
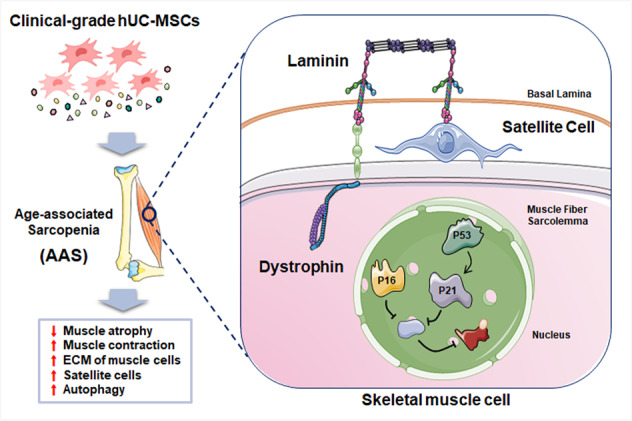
This study comprehensively evaluates the preclinical efficacy of clinical-grade hUC-MSCs in treating age-associated sarcopenia (AAS), and demonstrates that hUC-MSCs restore skeletal muscle strength and performance in two AAS mouse models via raising the expression of extracellular matrix proteins, activating satellite cells, enhancing autophagy, and impeding cellular aging, which highlights a promising strategy for AAS and other age-associated muscle diseases.

This study comprehensively evaluates the preclinical efficacy of clinical-grade hUC-MSCs in treating age-associated sarcopenia (AAS), and demonstrates that hUC-MSCs restore skeletal muscle strength and performance in two AAS mouse models via raising the expression of extracellular matrix proteins, activating satellite cells, enhancing autophagy, and impeding cellular aging, which highlights a promising strategy for AAS and other age-associated muscle diseases.

## Introduction

Sarcopenia is a progressive and generalized skeletal muscle disorder characterized by the degenerative loss of skeletal muscle strength and mass, involving the accelerated loss of muscle mass and function [1–3]. Studies have reported that aging appears to result in the disturbance of homeostasis in skeletal muscle and an imbalance of tissue regeneration, leading to an overall loss of skeletal muscle. Cellular changes in sarcopenic muscle include a reduction in the size and number of myofibers. This is due to the decreased number of fast myosin fibers with age, together with intramuscular and intermuscular fat infiltration, and a decreased number of satellite cells [4]. Sarcopenia could be divided into primary sarcopenia (or age-associated sarcopenia, AAS), caused by aging, and secondary sarcopenia (or disease-associated sarcopenia, DAS), caused by diabetes mellitus, cancer, chronic obstructive pulmonary disease, or heart failure [4–6], which also requires appropriate treatment of the underlying disease.

With the expansion of the aging population, the problem of AAS becomes increasingly severe, which seriously impacts the lifestyle of the elderly [7, 8]. Despite physical exercise is proven to be the most effective preventative measure for AAS in mouse models, it is often impractical or inefficient for elderly individuals with reduced functional capacities. Several clinical pharmaceuticals, such as testosterone, growth factors and dehydroepiandrosterone, have been reported to be helpful in treating AAS, but with controversial effects [3, 9]. Unfortunately, there are currently no approved therapies for AAS, making it imperative to develop new treatments.

Mesenchymal stem cells (MSCs) have gained widespread use in cell therapy due to their anti-fibrosis, immunomodulatory properties, as well as their ability to release various biologically active molecules [10–12]. It has been reported that MSCs could enhance muscular regeneration in animal and cellular models [13–18]. Particularly, human umbilical cord-derived mesenchymal stem cells (hUC-MSCs) are advantageous due to their higher yield without the invasive procedures or ethical issues, as well as their ability to secrete a wide range of multifunctional factors [19, 20]. Our group has previously developed a complete system for obtaining clinical-grade hUC-MSCs according to the current Good Manufacturing Practice (cGMP) guidelines. The hUC-MSCs obtained through this system have been shown to meet the quality criteria of the National Institute of Food and Drug Control (NIFDC) and possess good preclinical efficacy in the intervention or treatment of neurodegeneration associated with aging [21, 22]. Furthermore, therapeutic effects of hUC-MSCs have been demonstrated on muscular atrophy experimental models [15, 17, 23]. Although these studies lack a more comprehensive evaluation of preclinical efficacy, some positive effects bring new hope for the treatment of AAS. Therefore, we speculated that hUC-MSCs might be a superior source for reversing muscle dysfunction in AAS.

Here, for the first time, we comprehensively evaluated the preclinical efficacy of clinical-grade hUC-MSCs on two AAS mouse models, including the SAMP8 mice (a senescence-accelerated mouse commonly used as the AAS model) and D-galactose (D-gal)-induced aging model (a systemic and homogeneous aging model with the acceleration of senescence). Both models were proven to have the typical phenotype of AAS in our results. Based on the behavioral test, hematoxylin-eosin (H&E) staining, immunostaining and western blotting, we found that administration of hUC-MSCs effectively improved muscle strength, restored muscle morphology and performance of aging skeletal muscle in AAS mouse models. The mechanisms involved included raising the expression of crucial extracellular matrix proteins, activating skeletal satellite cells, enhancing autophagy, and impeding the cellular senescence by down-regulating p16/p53-p21 axis.

Collectively, our results demonstrated that hUC-MSCs transplantation could improve skeletal muscle dysfunction from multiple aspects, including cellular components, cell structure and cell function, and ultimately restore muscle strength in AAS mice. Additionally, our study clarified the underlying mechanism of systematically targeting AAS therapy, at least partially through reconstructing myocyte autophagy to provide self-energy supply and down-regulating the classic p16/p53-p21 axis to delay myocyte aging. More importantly, our study provided a promising strategy for the prevention and treatment of AAS and other age-associated muscle diseases.

## Results

### Clinical-grade hUC-MSCs improved muscle strength and restored skeletal muscle morphology both in SAMP8 mice and D-gal-induced aging mice

**Fig. 1:**
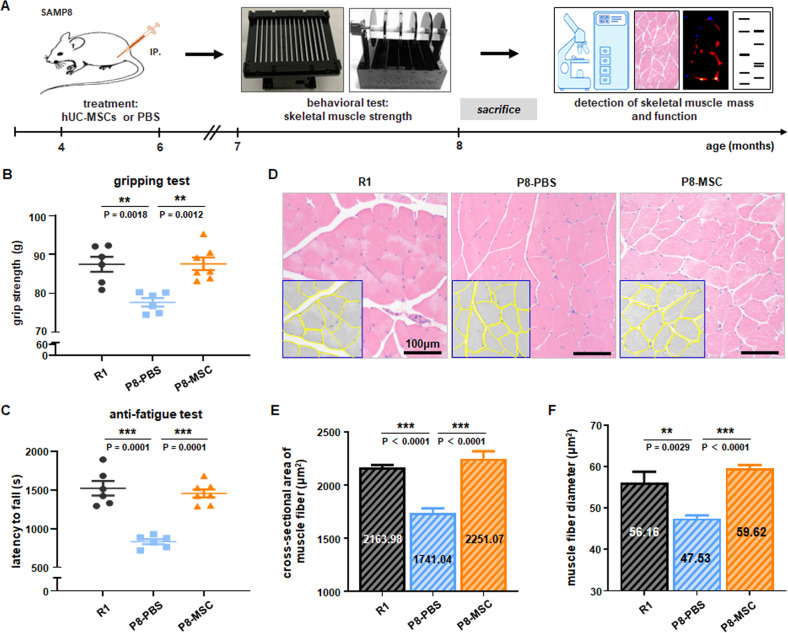
Clinical-grade hUC-MSCs improved muscle strength and restored skeletal muscle morphology in SAMP8 mice. **A** Illustrated in schematic form is the chronological sequence used for hUC-MSCs or PBS treatment, behavioral tests, immunostaining, western blotting and others. **B**, **C** Evaluation of muscle strength was performed by measuring gripping (*P*_*R1&PBS*_ = 0.0018; *P*_*MSC & PBS*_ = 0.0012) and anti-fatigue (*P*_*R1 & PBS*_ = 0.0001; *P*_*MSC & PBS*_ = 0.0001) capacities. The time of Latency to fall in the Rota Rod system was used to reflect the muscle endurance, and gripping test was used to show the grip strength of SAMP8 mice (*n* = 6–7 per group). **D** The representative cross-sections of gastrocnemius muscle were stained with H&E to observe better the morphology of muscle cells in R1, P8-PBS, and P8-MSC groups (scale bar = 100 μm). **E**, **F** Quantitative analysis of muscle fiber cross-sectional area (*P*_*R1 & PBS*_ < 0.0001; *P*_*MSC & PBS*_ < 0.0001) (μm^2^) and muscle fiber diameter (*P*_*R1 & PBS*_ = 0.0029; *P*_*MSC & PBS*_ < 0.0001) (μm^2^) in SAMP8 mice. (*n* = 8 or 10 views per group from 5–6 male mice; all data shown as mean ± SEM, *P < 0.05, **P < 0.01, ***P < 0.001).

Based on the detection of the cellular viability, morphology, differentiation potential and surface markers, the clinical-grade hUC-MSCs conformed to the quality standards of MSCs [21, 22] (Fig. S1). After treatment with hUC-MSCs, the behavior features of AAS mouse models were evaluated by grip test and anti-fatigue test, which are considered core metrics of sarcopenia [21, 22]. The results indicated that compared with the P8-PBS group, the mice treated with hUC-MSCs exhibited enhanced grip strength and anti-fatigue abilities (Fig. 1B, C), similar to the R1 group.

The mice were euthanasia subsequently (Figs. 1A, S2A), and the gastrocnemius muscles were collected and subjected to histopathological tests by cross section and vertical section [24]. To better characterise the muscle size on different treatment, we conducted H&E staining on the cross section of gastrocnemius muscle and measured the area and diameter of muscle cells. Initially, we observed a significant reduction in the cross-sectional area and diameter in the P8-PBS group, which were restored in the P8-MSC group. A similar treatment effect was noted in the D-gal-induced aging model (Fig. S2). These results demonstrated the treatment of hUC-MSCs could restore muscle functions and morphology in AAS mouse models.

### hUC-MSCs restored the ratio of slow and fast motor units of skeletal muscle in two mouse models

**Fig. 2:**
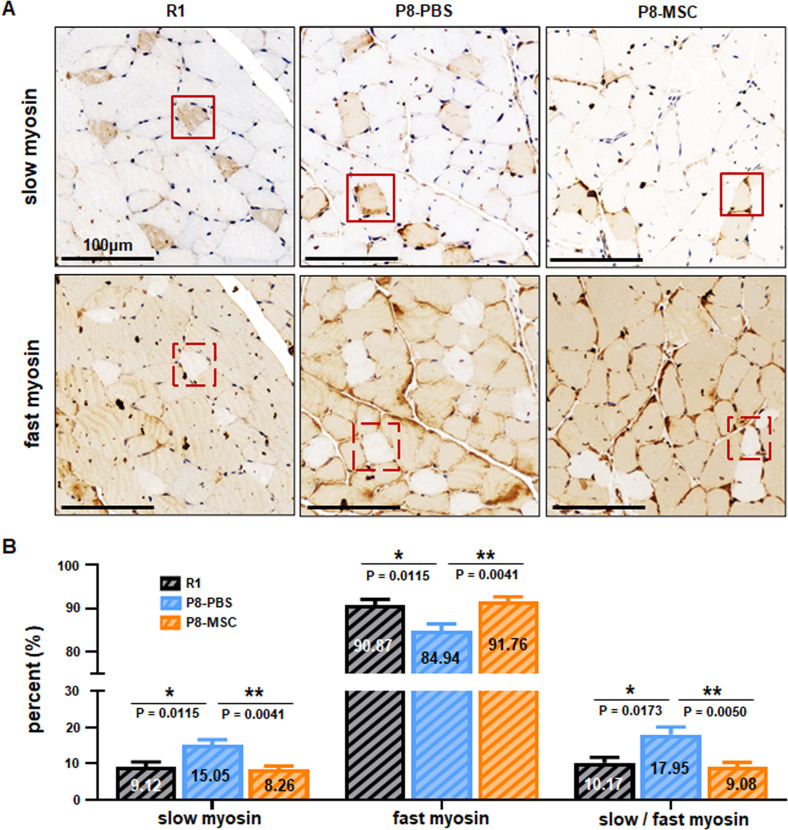
hUC-MSCs restored the ratio of slow and fast motor units of skeletal muscle in SAMP8 mice. **A** The representative immunohistochemical images of extensor digitorum longus (EDL) muscle cells illustrated the localization of fast myosin and slow myosin in R1, P8-PBS and P8-MSC mice. The square denoted the same muscle cell (scale bar = 100 μm). **B** The percentage of fast myosin and slow myosin in EDL muscle cells of SAMP8 mice (*P*_*R1 & PBS*_ = 0.0173; *P*_*MSC & PBS*_ = 0.0050) (*n* = 8 or 10 views per group from 5–6 male mice; all data shown as mean ± SEM, *P < 0.05, **P < 0.01, ***P < 0.001).

Skeletal muscle fibers can be classified into slow myosin (type I) fibers and fast myosin (type II) fibers [25], which play a critical role in accurately assessing the extent of muscle fiber impairment in aging muscle [26]. The primary cause of age-related loss of muscle mass is a decrease in the total number of both slow and fast myosin fibers, with the preferential atrophy of fast myosin fibers being secondary [27–29]. In order to estimate the potential of hUC-MSCs to prevent the slight decrease in fast fibers abundance, we examined the proportion of slow and fast myosin fibers in extensor digitorum longus (EDL). The EDL of skeletal muscle was then consecutively sectioned and stained for fast and slow muscle respectively. We observed that the unstained portion of the slow muscle section coincided with the deeply stained portion of the fast muscle section. Statistical results indicated that compared to the P8-PBS group, the ratio of slow and fast muscles in both the R1 group and P8-MSC group was lower (Fig. 2). Likewise, the same phenomenon of muscle contraction could also be found in the D-gal-induced aging model (Fig. S3). These results demonstrated that hUC-MSCs treatment restored the ratio of slow and fast motor units, thereby enhancing skeletal muscle performance.

### hUC-MSCs regulated the extracellular matrix of muscle cells in AAS mouse models

**Fig. 3:**
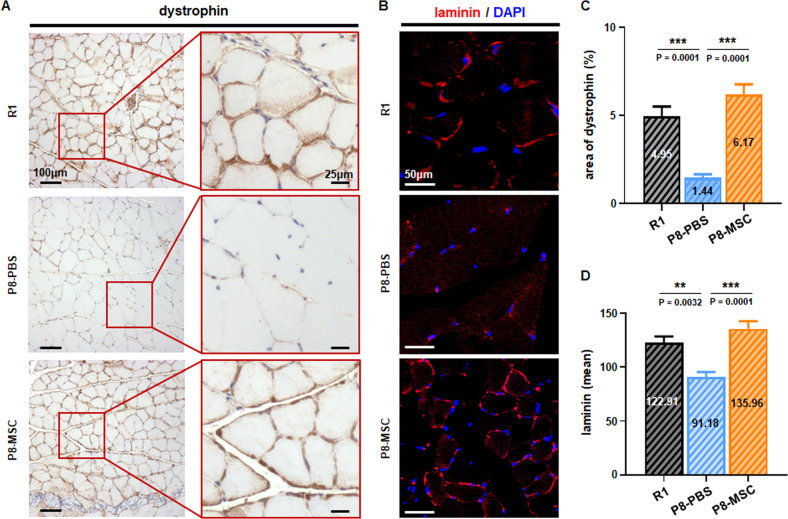
hUC-MSCs modulated the expression of important extracellular matrix proteins in SAMP8 mice. **A** The representative immunohistochemical images of ECM depicted dystrophin protein expression in the SAMP8 mouse model (scale bar = 100 μm), with individual cells expressing positive protein shown under high magnification within the square (scale bar = 25 μm). The percentage of dystrophin among different groups in the visual field area was quantified (*P*_*R1 & PBS*_ = 0.0001; *P*_*MSC & PBS*_ = 0.0001) (**C**) (*n* = 8 or 10 views per group from 5–6 male mice). **B** Representative immunohistochemistry images indicated the expression of Laminin protein in SAMP8 mice (scale bar = 50 μm). The average fluorescence value of Laminin protein expression was quantified according to the random visual field in different SAMP8 mice groups (*P*_*R1 & PBS*_ = 0.0032; *P*_*MSC & PBS*_ = 0.0001) (**D**) (*n* = 8 or 10 views per group from 5–6 male mice; all data shown as mean ± SEM, *P < 0.05, **P < 0.01, *** P < 0.001).

The extracellular matrix (ECM) plays a crucial role in the growth of muscle cells by creating the cellular niche and mediating the signal transduction [30]. With aging, the ECM surrounding muscle cells could undergo change, such as decreased stability of the muscle fiber sarcolemma (MFS) and weakened cell adhesion function [31]. In order to describe the changes in AAS models, we detected the expression of dystrophin and laminin, which played critical roles in stabilizing the sarcolemma of muscle fiber and participate in cell communication [31–34]. The immunofluorescent images showed that the muscle cells in SAMP8 mice exhibited lower levels of dystrophin and laminin proteins expression than those in the R1 mice. Following treatment with hUC-MSCs, the expression levels of dystrophin and laminin were noticeably increased, implying restoration of the muscle cells ECM (Fig. 3). Similarly, the elevated expressions of dystrophin and laminin in the D-gal-induced aging model were also observed after hUC-MSCs treatment (Fig. S4). These findings indicated that hUC-MSCs preserved muscle cell adhesion, improved their microenvironment and advanced muscle toughness and tensile strength in AAS by restoring the muscle ECM.

### hUC-MSCs restrained the decline in the number of muscle satellite cells in two mouse models

**Fig. 4:**
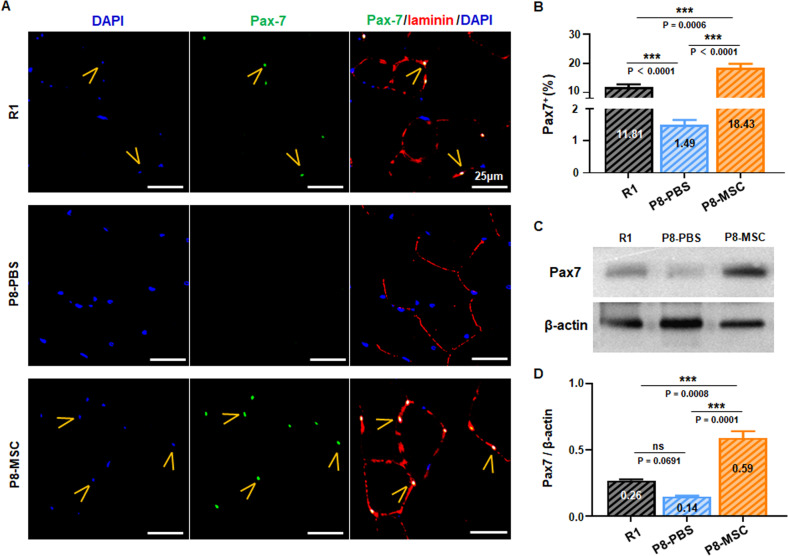
hUC-MSCs restrained the decline in the number of muscle satellite cells in SAMP8 mice. **A**, **B** Representative immunohistochemical images of satellite cells in skeletal muscle showed the expression of Pax-7, and the number of Pax-7^+^ cells in different visual fields in SAMP8 mice was calculated (*P*_*R1 & PBS*_ < 0.0001; *P*_*MSC & PBS*_ < 0.0001; *P*_*R1 & MSC*_ = 0.0006) (scale bar = 25 μm; *n* = 8 or 10 views per group from 5–6 male mice). **C**, **D** The expression of Pax-7 in gastrocnemius muscle of R1, P8-PBS and P8-MSC groups was detected by western blot and statistically analyzed (*P*_*R1 & PBS*_ = 0.0691; *P*_*MSC & PBS*_ = 0.0001; *P*_*R1 & MSC*_ = 0.0008) (*n* = 3 per group; all data shown as mean ± SEM, *P < 0.05, **P < 0.01, ***P < 0.001).

The decline in regenerative capacity of skeletal muscle with aging is attributed to the depletion and exhaustion of muscle stem cells (MuSCs), also known as satellite cells. To decipher whether hUC-MSCs could prevent this decline by reducing the depletion of MuSCs, we analyzed the expression of Pax7, the specific marker of MuSCs in skeletal muscle cells [35, 36]. Fluorescence image and Western blot revealed that the quantity of Pax-7^+^ cells in SAMP8 mice was lower than in R1 mice. Nevertheless, the number of MuSCs was significantly recovered after hUC-MSC treatment (Fig. 4). Moreover, in the D-gal-induced aging model, intraperitoneal injection of hUC-MSCs restored the number of MuSCs (Fig. S5). These results suggested that hUC-MSCs treatment can significantly recover the number of MuSCs in two AAS models, which maintained the stability of the stem cell pool, promoted proliferation and differentiation, and remodeled muscle fibers.

### hUC-MSCs increased autophagy and delayed muscle cells senescence via p16-Rb/p53-p21 axis

**Fig. 5:**
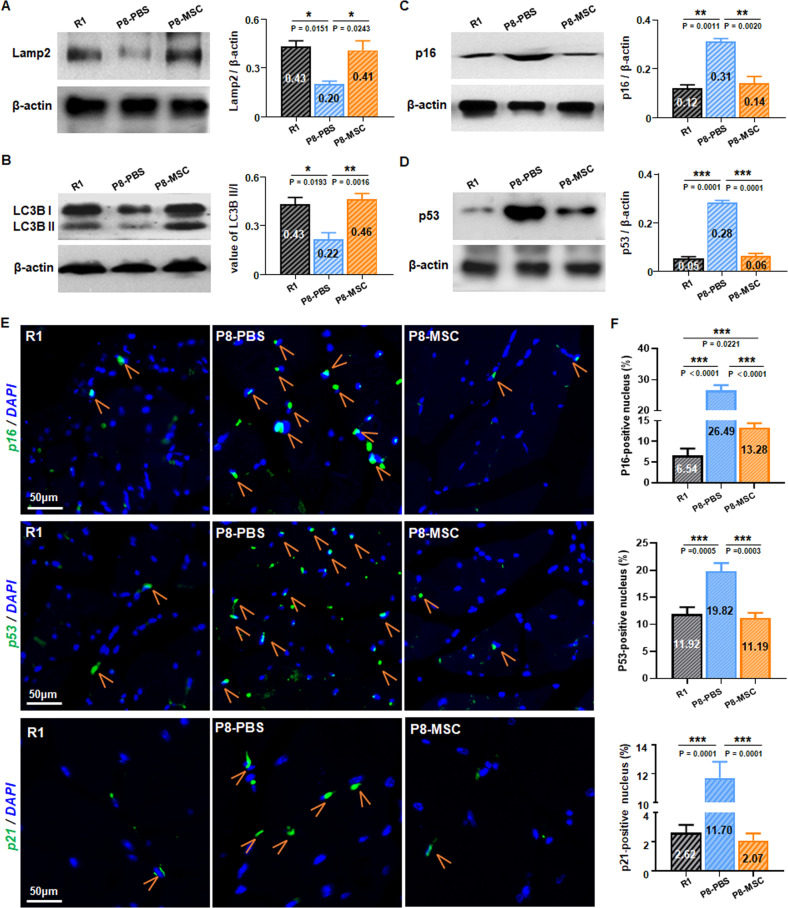
hUC-MSCs increased autophagy and delayed myocyte senescence through p16-Rb/p53-p21 axis in SAMP8 mice. **A**, **B**, **C**, **D** The expressions of Lamp2(*P*_*R1 & PBS*_ = 0.0151; *P*_*MSC & PBS*_ = 0.0243), LC3B II/I (*P*_*R1 & PBS*_ = 0.0193; *P*_*MSC & PBS*_ = 0.0016), p16(*P*_*R1 & PBS*_ = 0.0011; *P*_*MSC & PBS*_ = 0.0020) and p53(*P*_*R1 & PBS*_ = 0.0001; *P*_*MSC & PBS*_ = 0.0001) in muscle cells of R1, P8-PBS and P8-MSC mice were detected by western blot (n = 3 per group; all data shown as mean ± SEM, *P < 0.05, **P < 0.01, ***P < 0.001). **E**, **F** Representative immunofluorescence images showed the expression of p16(*P*_*R1 & PBS*_ < 0.0001; *P*_*MSC & PBS*_ < 0.0001; *P*_*R1 & MSC*_ = 0.0221), p53(*P*_*R1 & PBS*_ = 0.0005; *P*_*MSC & PBS*_ = 0.0003) and p21(*P*_*R1 & PBS*_ = 0.0001; *P*_*MSC & PBS*_ = 0.0001) in skeletal muscle in R1, P8-PBS and P8-MSC mice (scale bar = 50 μm) and quantified its expression based on the mean fluorescence value in various perspectives of view (*n* = 8 or 10 views per group from 5–6 male mice; all data shown as mean ± SEM, ***P < 0.001).

It is widely acknowledged that cellular autophagy is closely intertwined with senescence. Decreased autophagy may hasten the aging process, whereas increased autophagy holds the potential for anti-aging effects [37]. To investigate how hUC-MSCs could ameliorate the muscle dysfunctions in AAS mouse models by regulating muscle autophagy, we evaluated the expression levels of Lamp2 [38] and LC3- II/I [39], which are autophagy-related biomarkers. The western blot images showed the expression levels of Lamp2 and LC3- II/I in the P8-MSC group (Fig. 5A, B) and the D-gal-MSC group (Fig. S6A, B) were remarkably elevated as compared to the aging group, indicating that autophagy was stimulated in the presence of hUC-MSCs.

Several pathways, such as inhibition of p53, p16, and p21 activation, are implicated in the acceleration of senescence [40] and the inhibition of autophagy [41]. Western blot and immunofluorescence images demonstrated that the expression levels of p16, p53 and p21 were decreased in the P8-MSC group (Fig. 5C–F) and the D-gal-MSC group (Fig. S6C–F) as compared to the P8-PBS and the D-gal-PBS groups, respectively, suggesting that hUC-MSCs treatment could inhibit the activation of senescence-related pathways.

Collectively, hUC-MSCs played a pivotal role in revitalizing myocyte autophagy to furnish self-energy supply, and it could downregulate the classic p16 / p53-p21 axis to decelerate myocyte aging.

## Discussion

AAS, the primary clinical malady affecting the elderly, poses a significant challenge for achieving healthy aging [42]. It is estimated that approximately 5–13 % of the geriatric population, deemed “healthy”, is afflicted with AAS [43–45]. Furthermore, AAS is intrinsically linked to disabling conditions such as cognitive decline and osteoporosis [7, 46]. Despite the gravity of this condition, no viable therapies for AAS currently exist [47]. Hence, with the aging of the global populace accelerating, it is imperative to devise safe and effective intervention strategies to avert the transition to disability and medical institutionalization of older individuals [3].

Recent researches, including our own, have confirmed that hUC-MSCs were an ideal stem cell for universal application, and transplantation was an effective treatment for age-related degenerative diseases [48]. Furthermore, certain muscle atrophy models have reported the beneficial effects and effective utilization of hUC-MSCs in reducing muscle damage, enhancing tissue repair, and muscular regeneration, which might be based on their secretory capacity [13, 15–17, 49].

Selecting and confirming optimal experimental models was a key part of this study. To comprehensively assess the therapeutic effect of hUC-MSCs, two established aging mouse models, namely SAMP8 and D-gal-induced aging mice, were selected as subjects for investigation. SAMP8 mice are a frequently utilized animal model that exhibit age-associated muscle atrophy [48]. The age-related pathological phenotypes of SAMP8 during the aging process are comparable to those observed in elderly humans, such as senile amyloidosis, contracted kidney, and senile osteoporosis [50, 51]. SAMP8-induced muscle atrophy is predominantly characterized by fast type muscles, resembling symptoms of sarcopenia. Therefore, SAMP8 is the most commonly employed as an accelerated aging mouse model to study sarcopenia [42]. So, we used SAMP8 mice as an animal model to study sarcopenia and explore the potential of clinical-grade hUC-MSCs to improve skeletal muscle dysfunction in age-associated sarcopenia mice.

Another aging mouse model, D-gal-induced aging mice, was also incorporated in our study. The administration of D-galactose to these animals triggers several aging-related traits, such as reduced longevity, increased oxidative stress [52], decreased activity of antioxidant enzymes [53], mitochondrial DNA mutation [54] and mitochondrial dysfunction [55], which may also be correlated with skeletal muscle atrophy in aging. As such, the D-gal-induced aging mouse model is commonly used in research on senile diseases, anti-aging measures, and drug screening for sarcopenia-related fibrosis [56]. This model is advantageous due to its simplicity, short modeling time, and good repeatability. However, the phenotype of this model in AAS has not yet been revealed. Consequently, we observed and compared D-gal-induced aging mice with normal mice based on multiple indicators of AAS-related phenotypes observed in the SAMP8 model. Intriguingly, we verified for the first time that the D-gal-induced aging model exhibited typical AAS features (Figs. S2–S6), providing a supplementary preclinical research model for comprehensively screening clinical sarcopenia treatments.

Using these two animal models, we focused on evaluating the preclinical efficacy of hUC-MSCs in the treatment of AAS. The clinical diagnosis of sarcopenia typically starts with the measurement of muscle strength, that is, grip strength [4]. Our findings indicated that the hUC-MSCs-treated mice exhibited enhanced grip strength and anti-fatigue abilities in both SAMP8 mice (Fig. 1B, C) and D-gal-induced aging mice (Fig. S2B). Building on these results, we proceeded to analyze the pathological structure. One of the critical pathologies of AAS is age-related muscle mass loss owing to the decrease of MyHC fibers [57, 58] and the reduction of fast myosin fibers in the EDL [59]. At the cellular level, we observed that hUC-MSCs could effectively prevent atrophy of muscle fibers (Figs. 1D–F, S2C–E). These results suggested that hUC-MSCs treatment can significantly improve muscle strength and performance at the behavioral level in AAS mice.

With the above important findings, we focused on several core indicators that affect muscle mass and function during aging, such as slow and fast myosin fibers, ECM-related proteins, and muscle satellite cell status. Age-related loss of muscle mass was primarily due to a decrease in the total number of slow and fast myosin fibers and secondarily to the preferential atrophy of fast myosin fibers [27–29]. We examined the proportion of slow and fast myosin fibers in the EDL, and discovered that hUC-MSCs treatment restored the ratio of slow and fast motor units, thereby improving skeletal muscle mass in both mouse models (Figs. 2, S3). With aging, the ECM surrounding muscle cells could also change, resulting in reduced muscle fiber sarcolemma stability and weakened cell adhesion function [31]. We detected important ECM-related proteins dystrophin and laminin, which play critical roles in stabilizing muscle fiber’s sarcolemma and participate in cell communication [31–34]. The results demonstrated that hUC-MSCs treatment elevated the expression levels of dystrophin and laminin in both AAS mouse models (Figs. 3, S4), indicating that hUC-MSCs effectively promoted the establishment of ECM, providing a niche for signaling transduction between muscle cells [60, 61]. Preclinical studies have shown that during aging, there is a striking decline in skeletal muscle regenerative function, and muscle regeneration and repair can be facilitated by a population of dedicated MuSCs, also known as satellite cells, that reside in anatomically defined niches within muscle tissues and are generally characterized by expression of the myogenic transcription factor Pax7 [35, 36]. We then detected the expression level of Pax7 (the specific marker of satellite cells) and found that after hUC-MSCs treatment, the number of Pax7^+^ cells in AAS mice was significantly recovered (Figs. 4, S5), which proved hUC-MSCs could maintain the stability of the stem cell pool, or promote its proliferation and differentiation, and remodel muscle fibers.

After evaluating the protective effects of hUC-MSCs on skeletal muscle mass and function in SAMP8 and D-gal-induced aging mouse models, we further explored the underlying mechanisms that mediate the ability of hUC-MSCs to rejuvenate the physiological characteristics of age-associated sarcopenia. It is well known that cellular autophagy is closely related to senescence. With aging, a decline in autophagy can lead to decreased clearance of oxidatively damaged proteins and dysfunctional mitochondria, further feeding a cycle of cell damage and accelerating aging [41, 62]. Conversely, increased autophagy has the potential to combat aging [37]. Lamp2 and microtubule-associated protein light chain 3 (LC3) are common biomarkers associated with autophagy. LC3-I, the cytosolic form of LC3, is further converted to an autophagosome-associated form (LC3-II), so the LC3-II/LC3-I ratio is often used as an indicator to determine autophagic activity [38, 39]. To investigate whether hUC-MSCs could reverse the muscle dysfunctions in AAS mouse models via regulating muscle autophagy, we assessed the expression levels of Lamp2 and LC3- II/I ratio using western blotting analysis. Our results showed that compared with the respective PBS group, the expression levels of Lamp2 and LC3- II/I ratio increased significantly in the P8-MSC group (Fig. 5A, B) and the D-gal-MSC group (Fig. S6A, B) compared to their respective PBS group, indicating that autophagy was activated in the presence of hUC-MSCs.

Further, we investigated the signal pathways for the regulation of autophagic and aging signals in the presence of hUC-MSCs. Accelerated senescence and inhibited autophagy are associated with various pathways, such as inhibition of p53, p16 and p21 activation [40, 41]. Inhibition of the classical intracellular signal p16/p53-p21 axis [40] can up-regulate the expression of CDK cyclin and promote phosphorylation of Rb protein expression, thereby preventing further cell senescence, reducing the accumulation of damaged proteins, providing energy for the activation of MuSCs, and enabling muscle regeneration when was needed [63–67]. Specifically, our results demonstrated that the expressions of p16, p53 and p21 were decreased in the P8-MSC group (Fig. 5C–E) and the D-gal-MSC group (Fig. S6C–E) compared to their respective PBS group, suggesting that hUC-MSCs treatment could inhibit the activation of senescence-related pathways. Therefore, hUC-MSCs played a crucial role in reconstructing myocyte autophagy to provide self-energy supply, and could down-regulate the classic p16/p53-p21 axis to delay myocyte aging.

**Fig. 6:**
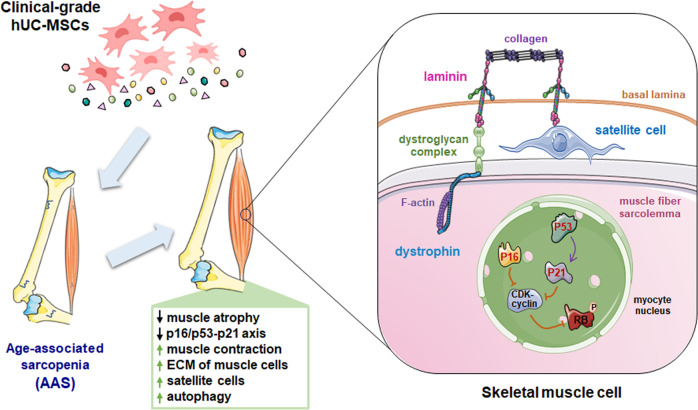
Schematic diagram of hUC-MSCs treatment in age-associated sarcopenia. hUC-MSCs release a variety of bioactive molecules to the skeletal muscle cells of AAS mice through the secretome. The satellite cells in the muscle cells are located between the basal lamina (BL) and the muscle fiber sarcolemma (MFS), where they interact with the matrix components of the niche. The satellite cells expressing Pax-7 at rest bind to collagen and laminin. In addition, MFS is connected to BL through the dystroglycan complex and laminin, and the dystrophin complex is combined with the F-actin cytoskeleton through dystrophin. Within the muscle nucleus, the secretory molecules of hUC-MSCs promote muscle autophagy and slow down aging by down-regulating the levels of p53, as well as its downstream molecules p21 and p16.

Our results collectively demonstrated that transplantation of hUC-MSCs could improve skeletal muscle dysfunction from multiple perspectives, encompassing cellular components, cell structure, and cell function. Ultimately, this restoration results in muscle strength recovery in AAS mice, as evidenced by Fig. 6. Despite these important findings, we believe there are still several interesting questions worthy of further exploration that necessitate well-designed protocols to validate. For instance, it is still unclear whether hUC-MSCs treatment promotes the “increase” of muscle satellite cells in AAS mice or prevents aging-induced satellite cell decline, both of which require additional research. Additionally, the aging process is incredibly intricate, we speculate that aside from improving the autophagy efficiency and reducing the expression of aging-related proteins (p16 and p21), hUC-MSCs infusion may regulate other aging-related proteins and pathways, playing a role in reversing muscle aging. More importantly, we will further promote the application of clinical-grade hUC-MSCs in the treatment of AAS. The administration route of hUC-MSCs, injection dose, administration time and other factors can significantly affect the residence time, viability, and homing of MSCs [68]. Therefore, we will further optimize the major variables in future experiments to ensure the therapeutic outcome in treating AAS is maximized and to promote clinical transformation.

## Conclusion

To our knowledge, this is the first study on applying clinical-grade hUC-MSCs to comprehensively evaluate the preclinical efficacy on two AAS mouse models. Furthermore, we established the D-gal-induced aging model as a potential candidate model for AAS, as it exhibits characteristic AAS features alongside the SAMP8 mice, a senescence-accelerated mouse model of AAS. Collectively, the data demonstrated that the clinical-grade hUC-MSCs could effectively counteract the progression of muscle aging in AAS mice, and clarified the underlying mechanism of systematically targeting AAS therapy. This therapy worked at least partially through reconstructing myocyte autophagy to provide self-energy supply and down-regulating the classic p16/p53-p21 axis to delay myocyte aging. As such, we propose that, hUC-MSC-based therapy may be a promising and effective muscle protective candidate to prevent AAS and other progressive age-related muscle diseases.

## Materials and methods

### Isolation, cultivation and identifition of hUC-MSCs

The procedures involving human subjects in this study were approved by the Ethics Committee at the Sun Yat-Sen Memorial Hospital (Approval number: 2021-01-01), and all patients provided their written informed consent to participate. The clinical-grade hUC-MSCs used in this study had been optimized in previous research, and the entire process, from isolation and cultivation to identification, quality control, and storage, was confirmed to meet the quality standards [69]. The generation time of hUC-MSCs used in this study was strictly limited to passages 3 to 5.

### Animals

The SAMP8 and SAMR1 mice were purchased from the Health Science Center of Peking University and were not tested until they reached four months of age. Additionally, 2-month-old male C57BL/6 mice were used for the study [69]. They were housed in a controlled environment with appropriate temperature and humidity levels, under a 12-h light/12-h dark cycle, and provided ad libitum access to food and tap water. After the experiment, the mice were euthanized using CO_2_ euthanized chamber. The Ethics of Animal Experiments Committee at the Academy of Military Medical Sciences approved all animal procedures, and all animals were cared for following the Guide for the Care and Use of Laboratory Animals.

### Treatment

For the SAMP8 mouse model, the SAMR1 mouse served as control group (R1 group); SAMP8 mice were divided into two groups: (I) P8-PBS group (*n* = 10–12); (II) P8-MSC group (*n* = 10-12), which were intraperitoneally injected with 5 × 10^6^ hUC-MSCs once a week for eight weeks. Accordingly, male C57BL/6 mice were divided into two groups at two months: normal (*n* = 10) and D-gal treatment (*n* = 26) groups. D-galactose (100 mg/kg, Sigma, USA, G0750) was injected subcutaneously every day for eight weeks. At the age of seven months, the latter group was further divided into two subgroups (*n* = 13 in each subgroup): (I) D-gal-PBS group; (II) D-gal-MSC group, which were intraperitoneally administered 5 × 10^6^ hUC-MSCs once every two weeks for 12 weeks. After administering the injections, behavioral experiments were conducted on the mice, followed by euthanasia and collection of gastrocnemius muscle and EDL for further tests.

### Rota Rod System

The rotary rod was used to assess the muscle endurance and balance ability of the mice. The experiment consisted of two days of training followed by one test day. On the training day, the speed of the instrument was set at 10 r/min, and the time was set to 300 s. Each mouse received three training sessions per day, with a 30-min rest interval between each session. If a mouse fell off the rod during training, it was placed back on the rotating stick. After two days of training, it was guaranteed that each mouse could be familiar with and move stably on the rotating stick before the test day. On the test day, the speed of the instrument increased uniformly from 5 r/min to 40 r/min, and then stabilized at 40 r/min for 300 s. The falling time of the mice was recorded as the fatigue tolerance time, which is also known as the incubation period. If a mouse remained on the rod for the entire 300 s, the time was recorded as 300 s.

### Grip test

Put the mouse in the center of the metal mesh, then grasp its tail and pull it gently backward until the mouse’s paws are released. The apparatus would register the maximum gripping strength of the mouse limbs, record the readings, repeat the process 5 times, and calculate the mean value of the three highest grasping forces as the overall grasping force.

### Immunohistochemical experiment

The mice were perfused transcardially with ice-cold saline, followed by 50 mL of 4 % paraformaldehyde (PFA), took out their muscle tissue, and fixed in 4 % PFA overnight. Each tissue was embedded in paraffin and sectioned coronally into 5-μm-thick slices. A standard histological immunohistochemical protocol was performed, which involved dewaxing and rehydration of the sections, incubating them with 3% H_2_O_2_ in methanol, and retrieving the antigen by placing the slides in target retrieval solution at 95 °C for 20 min. The sections were then incubated overnight with Monoclonal Anti-Myosin (Skeletal, Fast) antibody (Sigma, Germany, M4276), Monoclonal Anti-Myosin (Skeletal, Slow) antibody (Sigma, Germany, M8421), Rabbit polyclonal to Anti-Dystrophin (Abcam, USA, ab15277), or Anti-Myosin Heavy Chain Antibody (Sigma, Germany, 05-716) at 4 °C. After incubation with secondary antibody at room temperature for 30 min, the slides were incubated with HRP-peroxidase complex at room temperature for 30 another minutes. Reaction products were visualized using 3,3-diaminobenzidine (DAB) for counterstaining.

### Immunofluorescent staining

The sections of the gastrocnemius muscle were subjected to dewaxing and rehydration, followed by fixation in pre-cooled acetone at 4 °C for 25 min, and permeabilization in PBS containing 0.1% Triton X-100 for 15 min. The sections were then blocked for 1 h with 10 % normal Donkey Serum, before incubation with Rabbit polyclonal to Anti-Laminin (Abcam, USA, ab11575) or Anti-Pax7 Rabbit pAb antibody (Servicebio, China, GB113190) and Anti-p21 Rabbit pAb (Servicebio, China, GB11153). Incubation was carried out overnight at 4 °C followed by washing with PBS three times and incubation with the secondary antibody (Invitrogen, USA) for one hour at room temperature, followed by another round of washing. The nucleus was observed using 2 μM DAPI (Servicebio, China, G1012).

### Hematoxylin-Eosin staining

The sections of the gastrocnemius muscle underwent dewaxing and rehydration procedures, followed by hematoxylin staining for a duration of 10 min. The sections were then washed with running water, differentiated with 0.7% hydrochloric acid and ethanol for a few seconds, and rinsed with tap water. After 15 min, the sections were stained with 95% ethanol, alcohol-based eosin, 95% ethanol (I, II), 100% ethanol (I, II), xylene and xylene (I, II) for 30 seconds each, and finally covered a film. H&E staining was used for distinguishing skeletal muscle (red).

### Western blot analysis

Western blots were performed and analyzed as previously described [22]. Briefly, gastrocnemius muscle samples were homogenized in RIPA buffer, and then centrifuged at 12000 × g for 30 min at 4 °C. The quantification of protein in supernatant was accomplished using a BCA kit (Beyotime Biotechnology, Wuhan, China). The protein samples were boiled in the presence of sample buffer at 95 °C for 3 min. The proteins were subjected to the separation by sodium dodecyl sulfate polyacrylamide gel electrophoresis (SDS-PAGE), and subsequently transferred onto a nitrocellulose membrane. The target protein was probed by a corresponding antibody and then visualized by enhanced chemiluminescence (ECL) reagent and imaged by the chemiluminescence imaging system Amersham Imager 680 (General Electric Company, USA). Antibodies used for Western blots were: Anti-Pax7 (Abcam, USA, ab199010), Anti-alpha smooth muscle Actin (Abcam, USA, ab5694), Anti-LAMP2-Lysosome Marker (Abcam, USA, ab25631), Anti-Beta actin (Abcam, USA, ab8226), Anti-Cleaved LC3B (Sigma, Germany, L7S43), Anti-ARPC5/p16 ARC (Abcam, USA, ab51243), p53 (1C12) Mouse mAb (Cell Signaling Technology, USA, 1C12). Origin images of all western blot have been uploaded as a single ‘Supplemental Material’ file.

### Quantitative and statistical analysis

The cell density, nuclear area, cell area, and antibody expression in Immunohistochemical experiment, Immunofluorescent staining and Hematoxylin-Eosin staining were acquired via images by Tissue FAXS (Tissue Gnostics GmbH, Vienna Austria) with a Zeiss Axio Imager Z2 Microscope System at ×200 magnification. The cross-sectional area of 500 selected skeletal muscle fibers in the stained sections was measured and calculated using ImageJ software (version 1.8.0, National Institutes of Health) and Image-Pro Plus (version 6.0.0, media cybernetics).

Statistical analyses were executed with GraphPad Prism 9.0 software (GraphPad Software, San Diego, CA, USA) and presented as the mean value accompanied by the plus or minus standard error of the mean. The statistical significance of the differences among the three groups was determined using one-way analysis of variance (ANOVA), followed by Tukey post hoc tests, as depicted in the bar graph. A value of *P* <0.05 was considered to be statistically significant.

## Supplementary information

CDDIS-22-3682RR. Supplemental Figure Legends

Figure S1

Figure S2

Figure S3

Figure S4

Figure S5

Figure S6

CDDIS-22-3682RR. Original Western Blots

CDDIS-22-3682RR. The reproducibility checklist

## Data Availability

The data that support the findings of this study are available from the corresponding author upon reasonable request.
