# Supplementary material for: Clinical-grade human umbilical cord-derived mesenchymal stem cells improved skeletal muscle dysfunction in age-associated sarcopenia mice
Source: Cell Death Dis. 2023 May 12;14(5):321. doi: 10.1038/s41419-023-05843-8 (PMC10182022; doi:10.1038/s41419-023-05843-8)
Supplement: Supplementary file 1 — CDDIS-22-3682RR. Supplemental Figure Legends [file 41419_2023_5843_MOESM1_ESM.doc]

**Supplemental Figure Legends**

**Figure S1.** **Characteristics, differentiation and cell viability potential of clinical-grade hUC-MSCs.**

**(A) The markers of hUC-MSCs were analyzed by flow cytometry. Mesenchymal lineage markers (CD73, CD90 and CD105) were positive, hematopoietic and endothelial markers (CD34, CD45, CD19 and CD14) were negative, and HLA-DR was also negative. (B) Differentiation potential of hUC-MSCs into mesodermal lineages. The representative images of hUC-MSCs differentiating into adipocytes, osteocytes and chondrocytes are shown in the figure. Fat droplets were stained with Oil red O. Calcium phosphate deposits were stained with ALP and Alizarin Red. Proteoglycans with Toluidine Blue and Alcian Blue. (C) Cell viability analysis of clinical-grade hUC-MSCs of different generations.**

**Figure S2. hUC-MSCs improved muscle strength and restored skeletal muscle morphology in D-gal-induced mice.**

**(A)** The timeline of D-galactose injection, hUC-MSCs treatment, behavior tests, immunostaining, western blotting, and other procedures is illustrated in the schematic. **(B)** The time of Latency to fall in the Rota Rod system was used to evaluate the muscle endurance (*P_Nor & PBS_* =0.0055; *P_MSC & PBS_* =0.0004), and gripping test was used to show the muscle strength (*P_Nor&PBS_*=0.0486; *P_MSC&PBS_*=0.0398) in the D-gal-induced aging model. **(C)** The representative cross-sections of gastrocnemius muscle stained with H&E were examined to better visualize the morphology of muscle cells (scale bar=100μm). And a representative image of the ultrastructure of muscle cells obtained under an electron microscope is presented (scale bar=500 nm). **(D)** Skeletal muscle mass (*P_Nor & PBS_* =0.0016; *P_MSC & PBS_* =0.0002) and skeletal muscle index (*P_Nor & PBS_* =0.0208; *P_MSC & PBS_* =0.0021) were evaluated in D-gal-induced aging model (n=2 per group; all data shown as mean ± SEM, **P* < 0.05, ***P* < 0.01, ****P* < 0.001). (E) Quantitative analysis of muscle fiber cross-sectional area (*P_Nor & PBS_* <0.0001; *P_MSC & PBS_* <0.0001) (μm^2^) and muscle fiber diameter (*P_Nor & PBS_* <0.0001; *P_MSC & PBS_* <0.0001) (μm^2^) in D-gal-induced mice (n=8 or 10 views per group from 5-6 male mice; all data shown as mean ± SEM, **P* < 0.05, ***P* < 0.01, ****P* < 0.001).

**Figure S3. hUC-MSCs restored the ratio of slow and fast motor units of skeletal muscle in D-gal-induced mice.**

1. Representative immunohistochemical images of EDL muscle cells showed the location of fast myosin and slow myosin in the D-gal-induced aging model (scale bar=100μm)**.** (B) The percent of fast myosin and slow myosin in EDL skeletal muscle cells of different groups (*P_Nor & PBS_* =0.0001; *P_MSC & PBS_* =0.0001) (n=8 or 10 views per group from 5-6 male mice; all data shown as mean ± SEM, **P* < 0.05, ***P* < 0.01, ****P* < 0.001).

**Figure S4. hUC-MSCs modulated the expression of important extracellular matrix proteins in D-gal-induced mice.**

1. Representative immunohistochemical images of ECM showed dystrophin protein expression in D-gal-induced mice (scale bar=100μm). Individual cells expressing positive protein under high magnification were displayed in the square (scale bar=25 μm). The percentage of dystrophin among different groups in the visual field area was quantified (*P_Nor & PBS_* =0.0008; *P_MSC & PBS_* =0.0014) (C) (n=8 or 10 views per group from 5-6 male mice). (B) Representative immunohistochemistry images illustrated Laminin protein expression (scale bar=50μm). The average fluorescence value of Laminin protein expression was quantified according to the random visual field in different groups (*P_Nor & PBS_* =0.0003; *P_MSC & PBS_* =0.0059) (D) (n=8 or 10 views per group from 5-6 male mice; all data shown as mean ± SEM, **P* < 0.05, ***P* < 0.01, ****P* < 0.001).

**Figure S5. hUC-MSCs restrained the decline in the number of muscle satellite cells in D-gal-induced mice.**

(A, B) Representative immunohistochemical images of satellite cells in skeletal muscle demonstrated the expression of Pax-7, and the number of Pax-7^+^ cells in different visual fields in D-gal-induced mice was calculated (*P_Nor & PBS_* =0.0035; *P_MSC & PBS_* =0.0001; *P_Nor & MSC_* =0.0049) (scale bar=50μm; n=8 or 10 views per group from 5-6 male mice). (C, D) The expression of Pax-7 in gastrocnemius muscle of the Nor, D-gal-PBS and D-gal-MSC groups was detected by western blot and statistically analyzed (*P_Nor & PBS_* =0.0893; *P_MSC & PBS_* =0.0001; *P_Nor & MSC_*=0.0049) (n=3/per group; all data shown as mean ± SEM, **P* < 0.05, ***P* < 0.01, ****P* < 0.001).

**Figure S6. hUC-MSCs increased autophagy and delayed myocyte senescence in D-gal-induced ageing model through p16-Rb / p53-p21 axis.**

(A, B, C, D) The expressions of Lamp2(*P_Nor & PBS_* =0.0009 ; *P_MSC & PBS_* =0.0020), LC3B II/I(*P_Nor & PBS_* =0.0453; *P_MSC & PBS_* =0.0003), p16(*P_Nor & PBS_* =0.0001; *P_MSC & PBS_* =0.0007) and p53(*P_Nor & PBS_* =0.0001; *P_MSC & PBS_* =0.0004) in muscle cells of the Nor, D-gal-PBS and D-gal-MSC groups were detected by western blot (n=3 per group; all data shown as mean ± SEM, **P* < 0.05, ***P* < 0.01，****P* < 0.001). (E, F) Representative immunofluorescence images showed the expression of p16 (*P_Nor & PBS_* <0.0001; *P_MSC & PBS_* <0.0001), p53 (*P_Nor & PBS_* <0.0001; *P_MSC & PBS_* <0.0001) and p21 (*P_Nor & PBS_* =0.0001; *P_MSC & PBS_* =0.0001) in skeletal muscle in the Nor, D-gal-PBS and D-gal-MSC mice (scale bar=50μm) and its expression was quantified based on the mean fluorescence value in different perspectives of view (n=8 or 10 views per group from 5-6 male mice; all data shown as mean ± SEM, ****P* < 0.001).
