# Supplementary figures and images for: Clinical-grade human umbilical cord-derived mesenchymal stem cells improved skeletal muscle dysfunction in age-associated sarcopenia mice
Source: Cell Death Dis. 2023 May 12;14(5):321. doi: 10.1038/s41419-023-05843-8 (PMC10182022; doi:10.1038/s41419-023-05843-8)

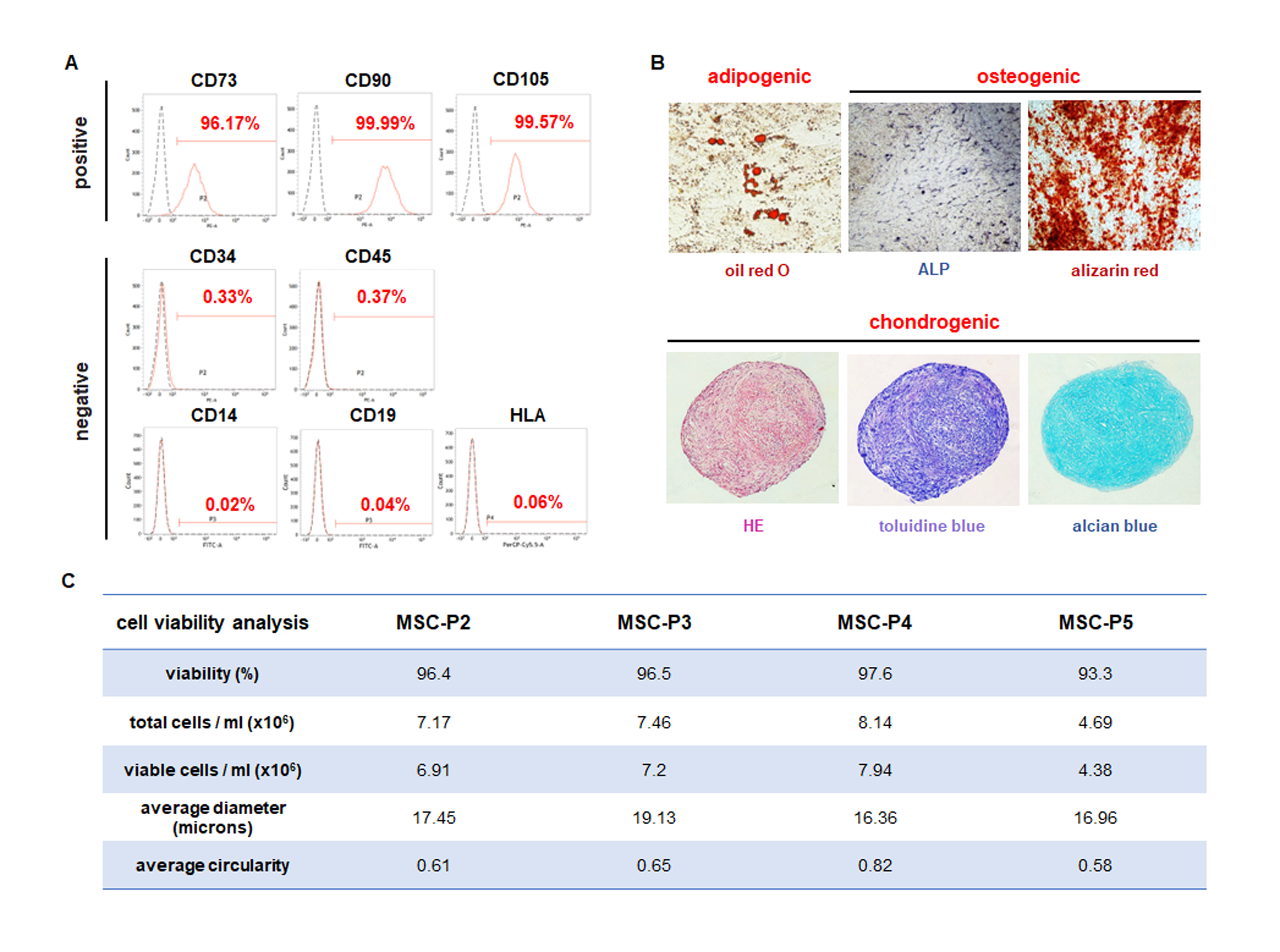

Supplement: Supplementary file 2 — Figure S1 [file 41419_2023_5843_MOESM2_ESM.tif]

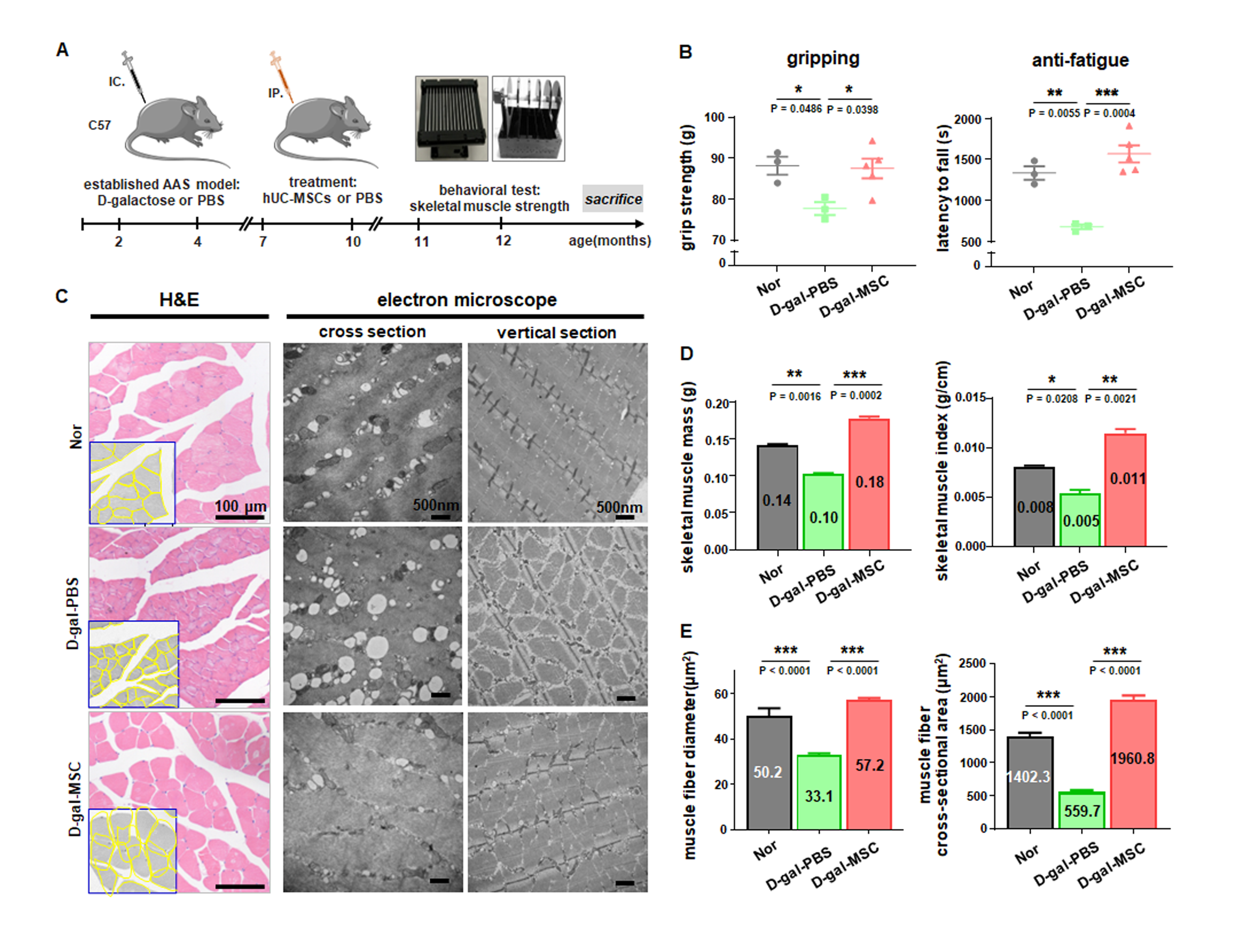

Supplement: Supplementary file 3 — Figure S2 [file 41419_2023_5843_MOESM3_ESM.tif]

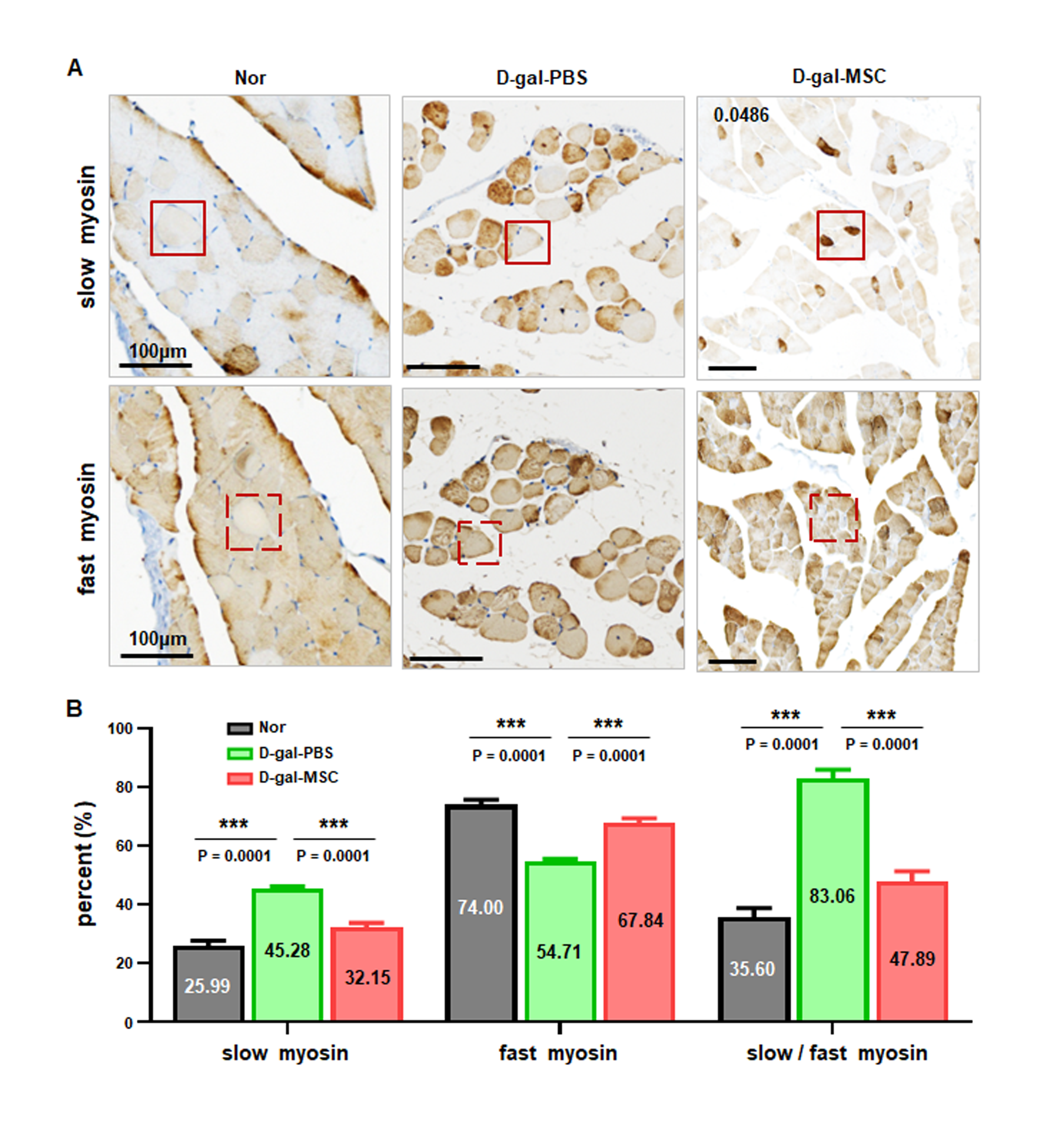

Supplement: Supplementary file 4 — Figure S3 [file 41419_2023_5843_MOESM4_ESM.tif]

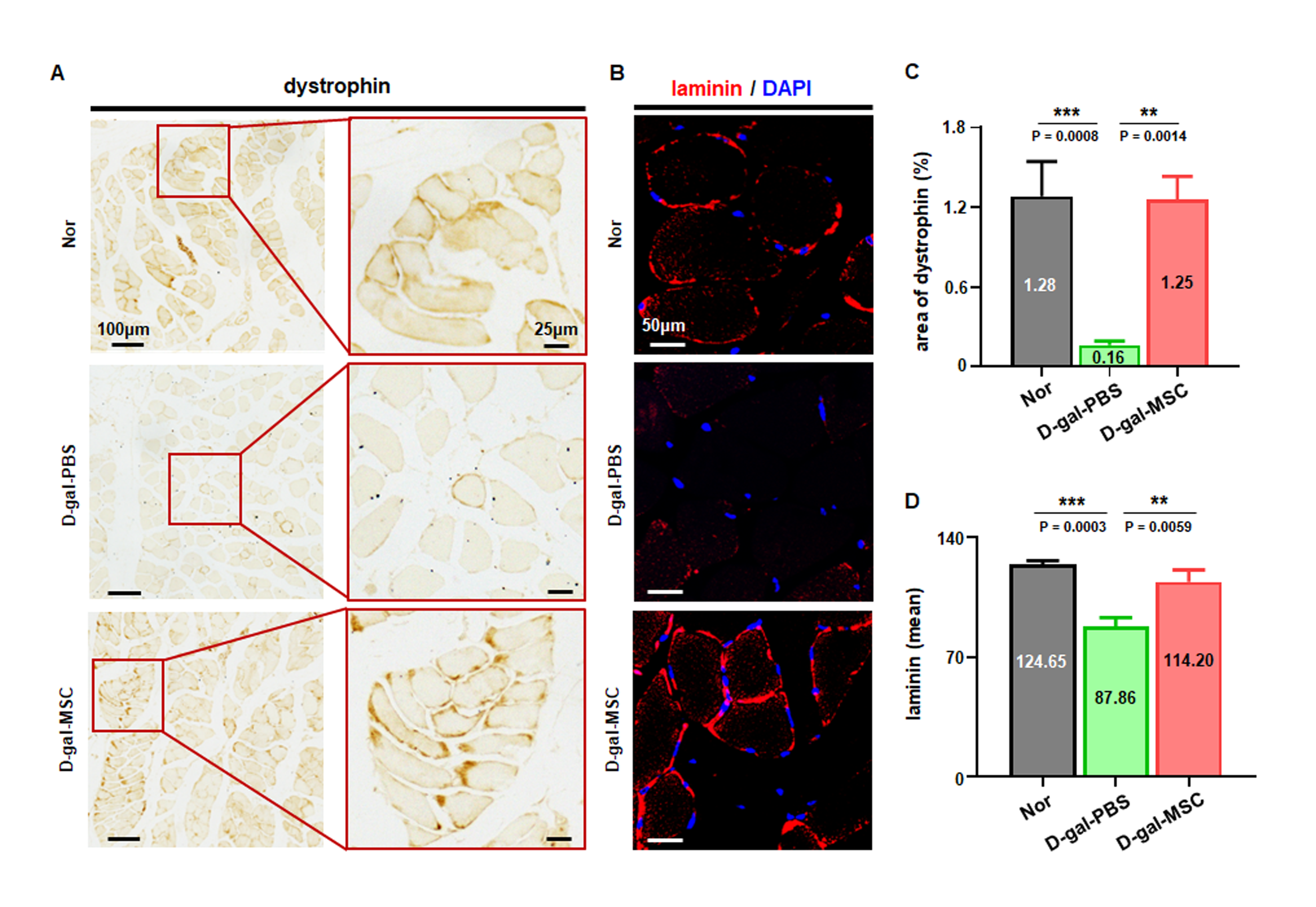

Supplement: Supplementary file 5 — Figure S4 [file 41419_2023_5843_MOESM5_ESM.tif]

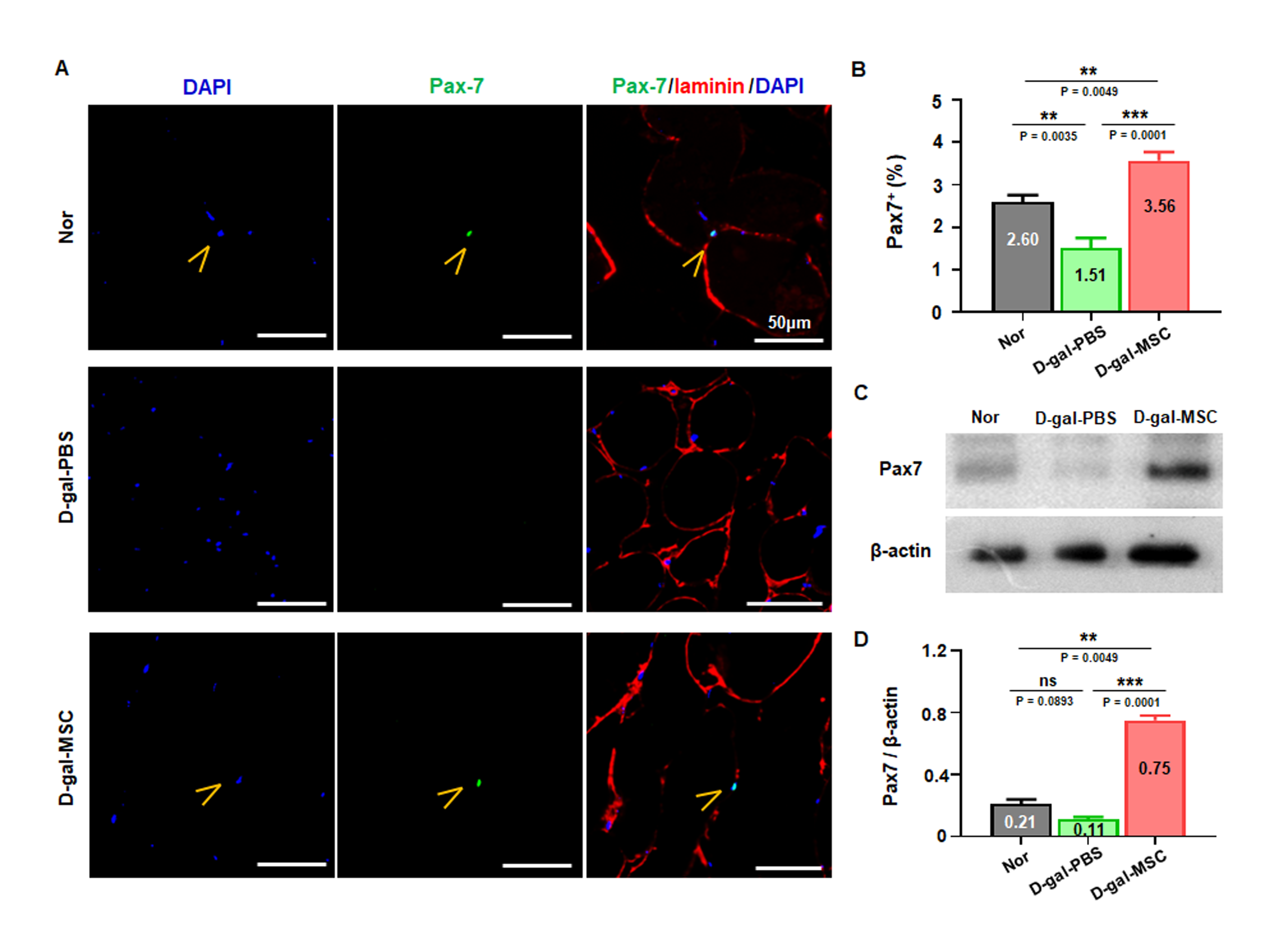

Supplement: Supplementary file 6 — Figure S5 [file 41419_2023_5843_MOESM6_ESM.tif]

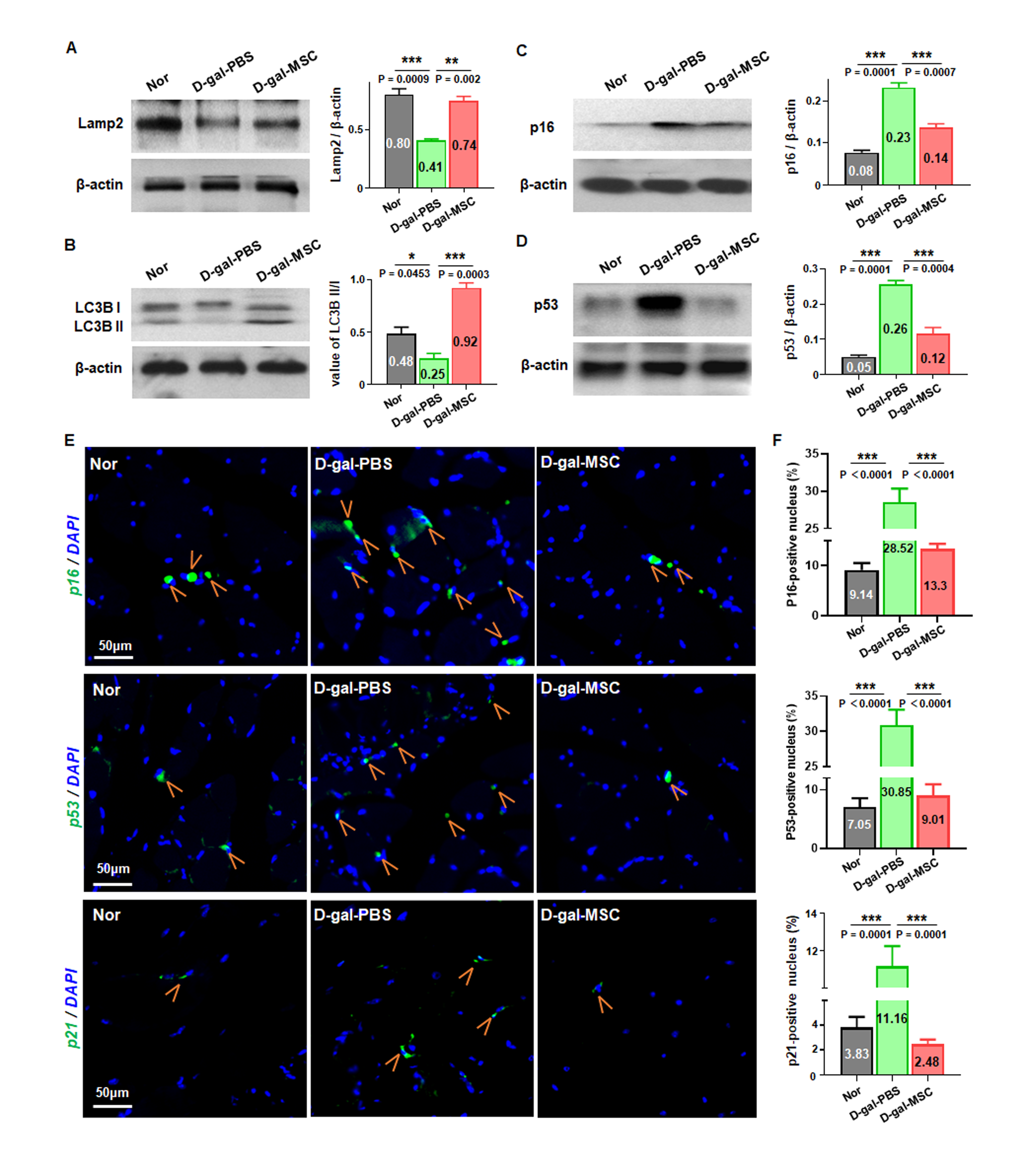

Supplement: Supplementary file 7 — Figure S6 [file 41419_2023_5843_MOESM7_ESM.tif]
