# Supplementary material for: Clinical-grade human umbilical cord-derived mesenchymal stem cells improved skeletal muscle dysfunction in age-associated sarcopenia mice
Source: Cell Death Dis. 2023 May 12;14(5):321. doi: 10.1038/s41419-023-05843-8 (PMC10182022; doi:10.1038/s41419-023-05843-8)

CDDIS-22-3682R

Clinical-Grade Human Umbilical Cord-Derived Mesenchymal Stem Cells Improved Skeletal Muscle Dysfunction in Age-Associated Sarcopenia Mice

Original Western Blots

- Note: The representative results of each indicator were listed in the first column.

Figure 4. hUC-MSCs restrained the decline in the number of muscle satellite cells in SAMP8 mice.

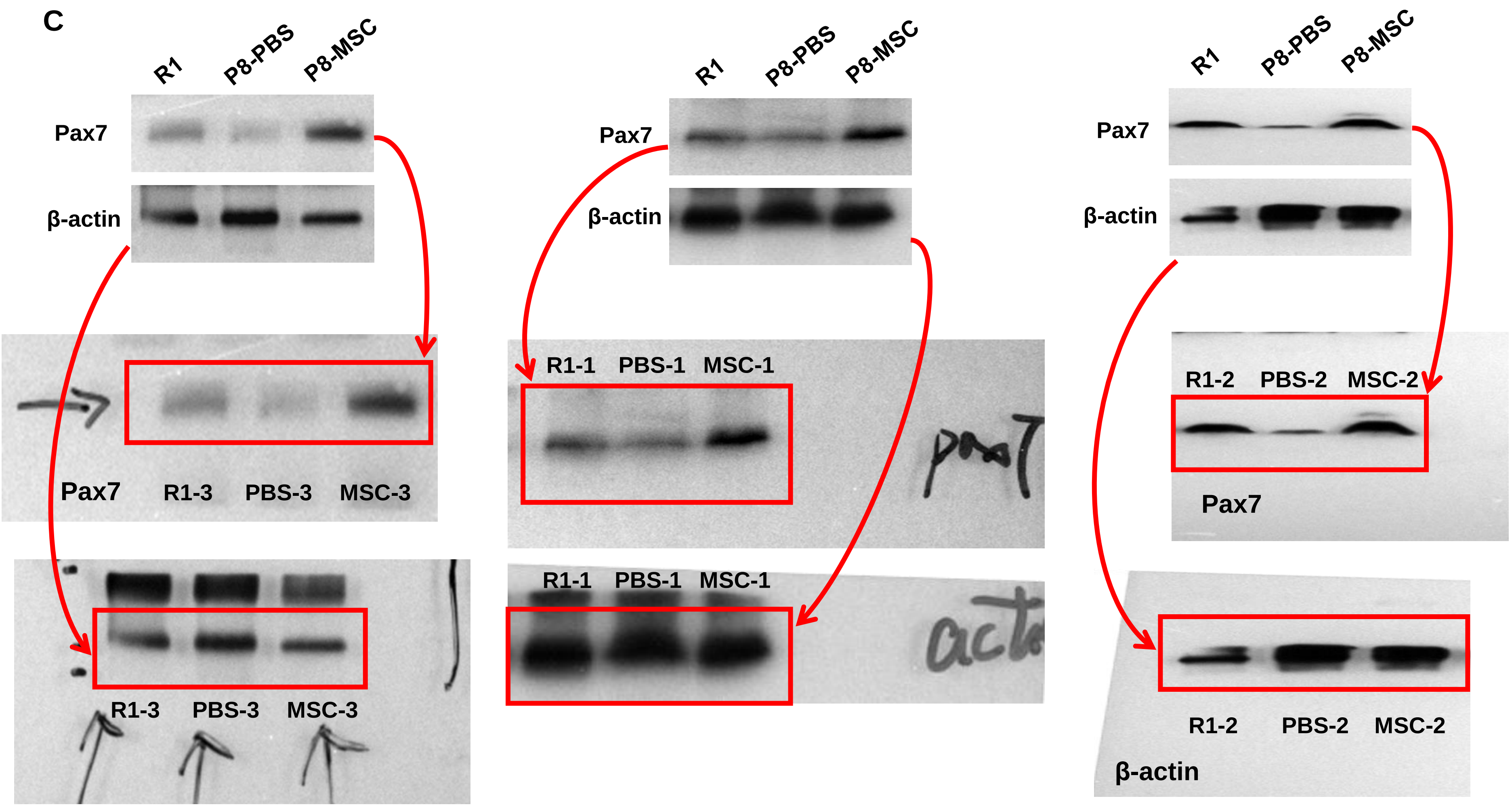

Figure S4. hUC-MSCs modulated the expression of important extracellular matrix proteins in D-gal-induced mice.

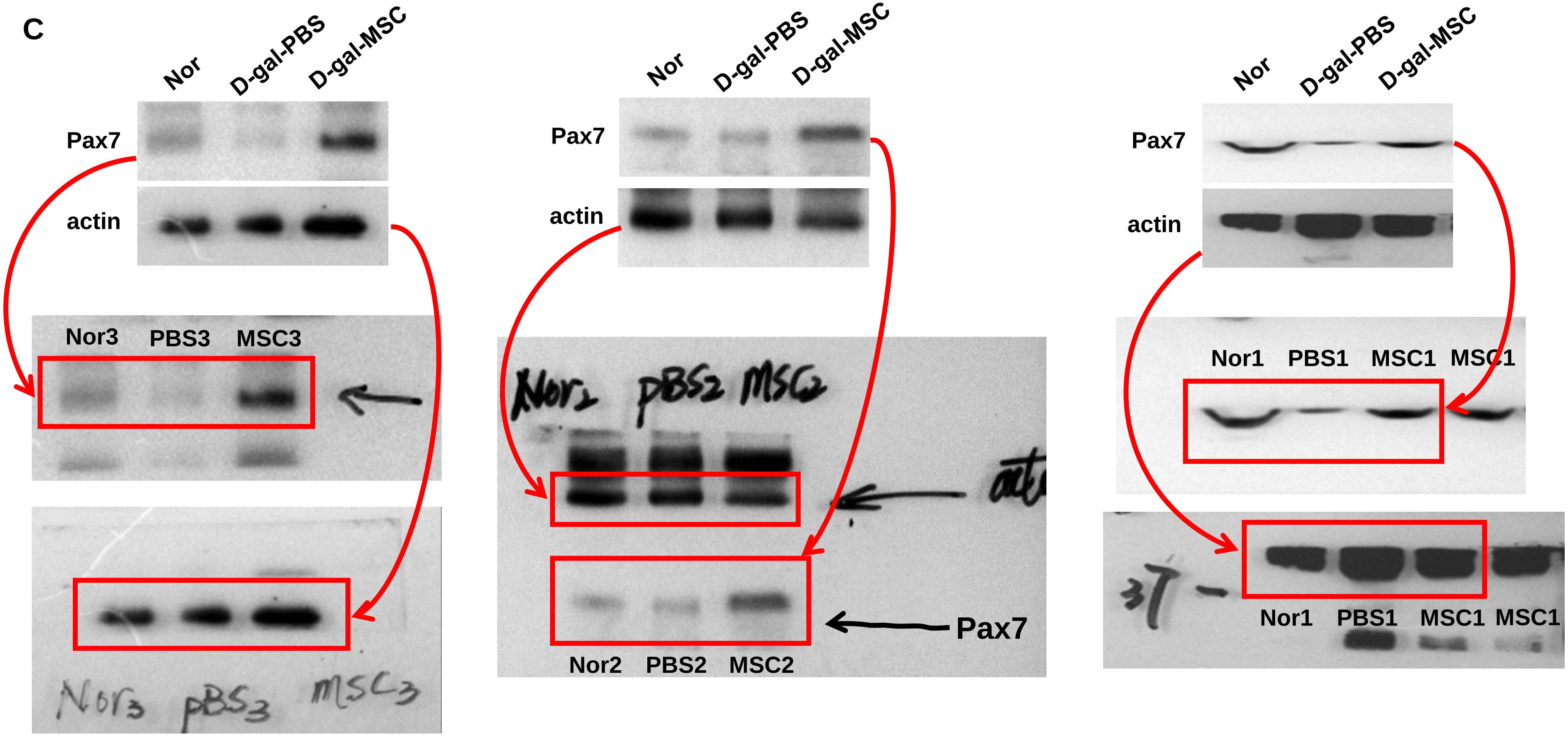

Figure 5. hUC-MSCs increased autophagy and delayed myocyte senescence through p16-Rb / p53-p21 axis in SAMP8 mice.

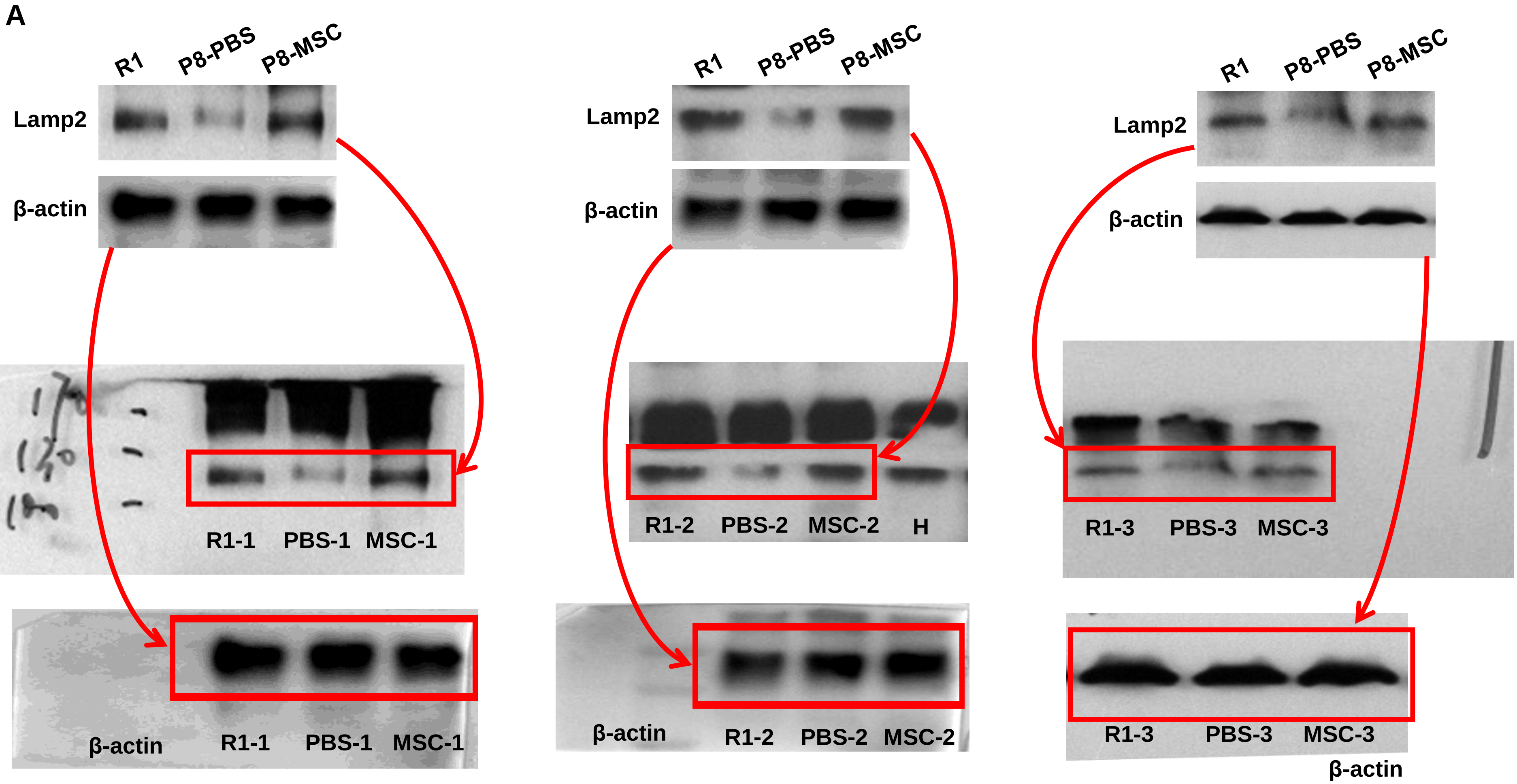

Figure 5. hUC-MSCs increased autophagy and delayed myocyte senescence through p16-Rb / p53-p21 axis in SAMP8 mice.

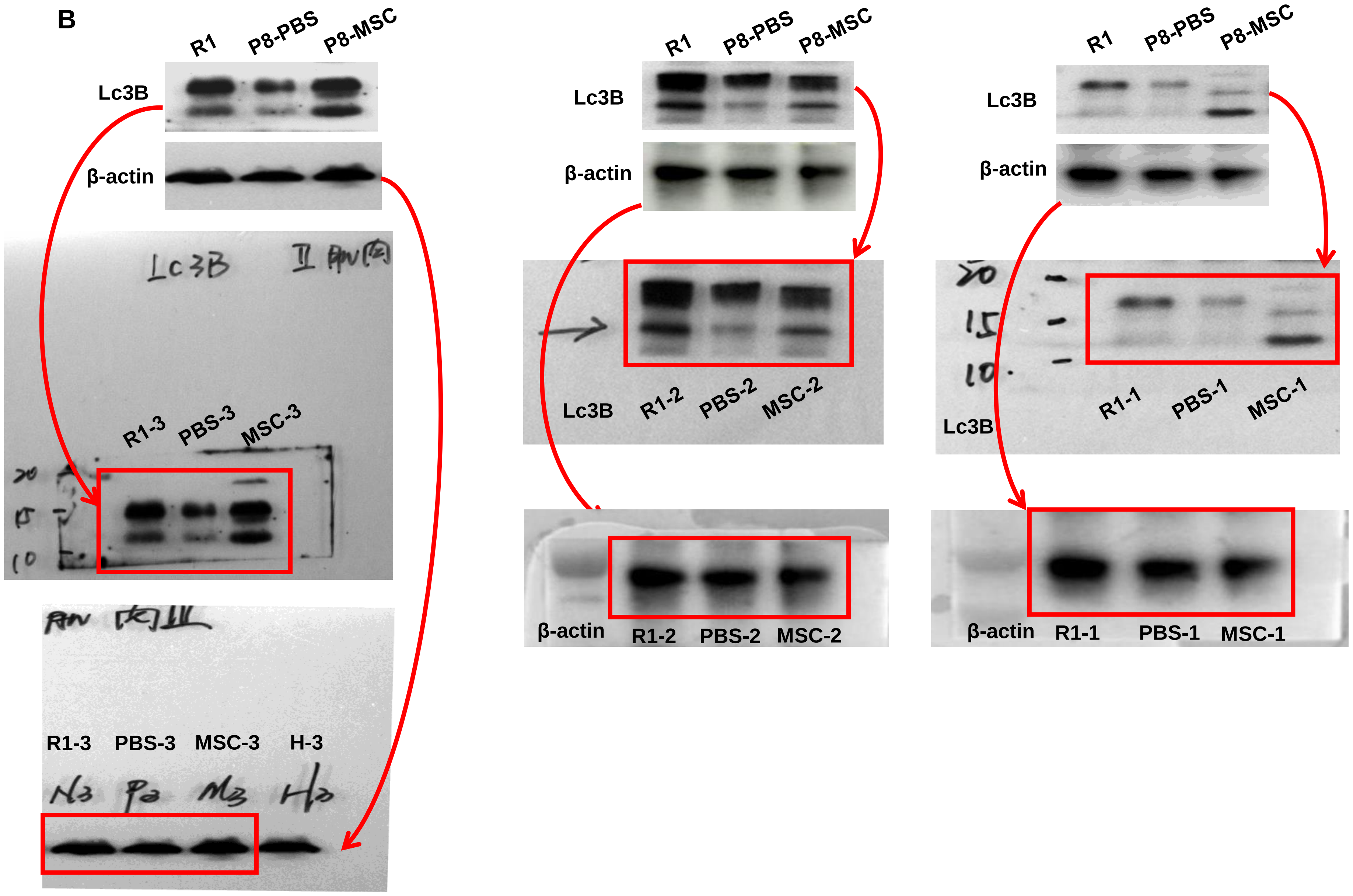

Figure 5. hUC-MSCs increased autophagy and delayed myocyte senescence through p16-Rb / p53-p21 axis in SAMP8 mice.

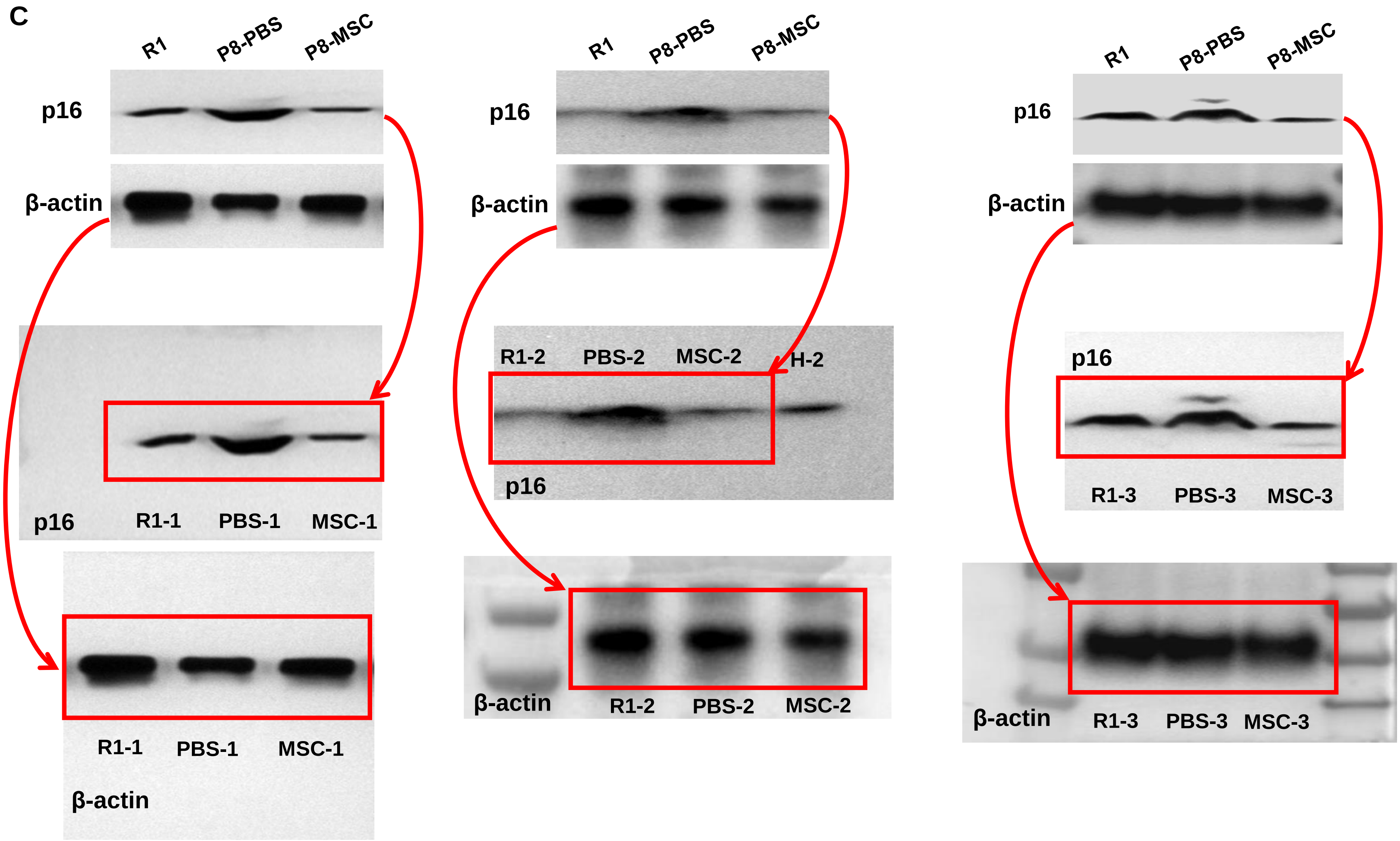

Figure 5. hUC-MSCs increased autophagy and delayed myocyte senescence through p16-Rb / p53-p21 axis in SAMP8 mice.

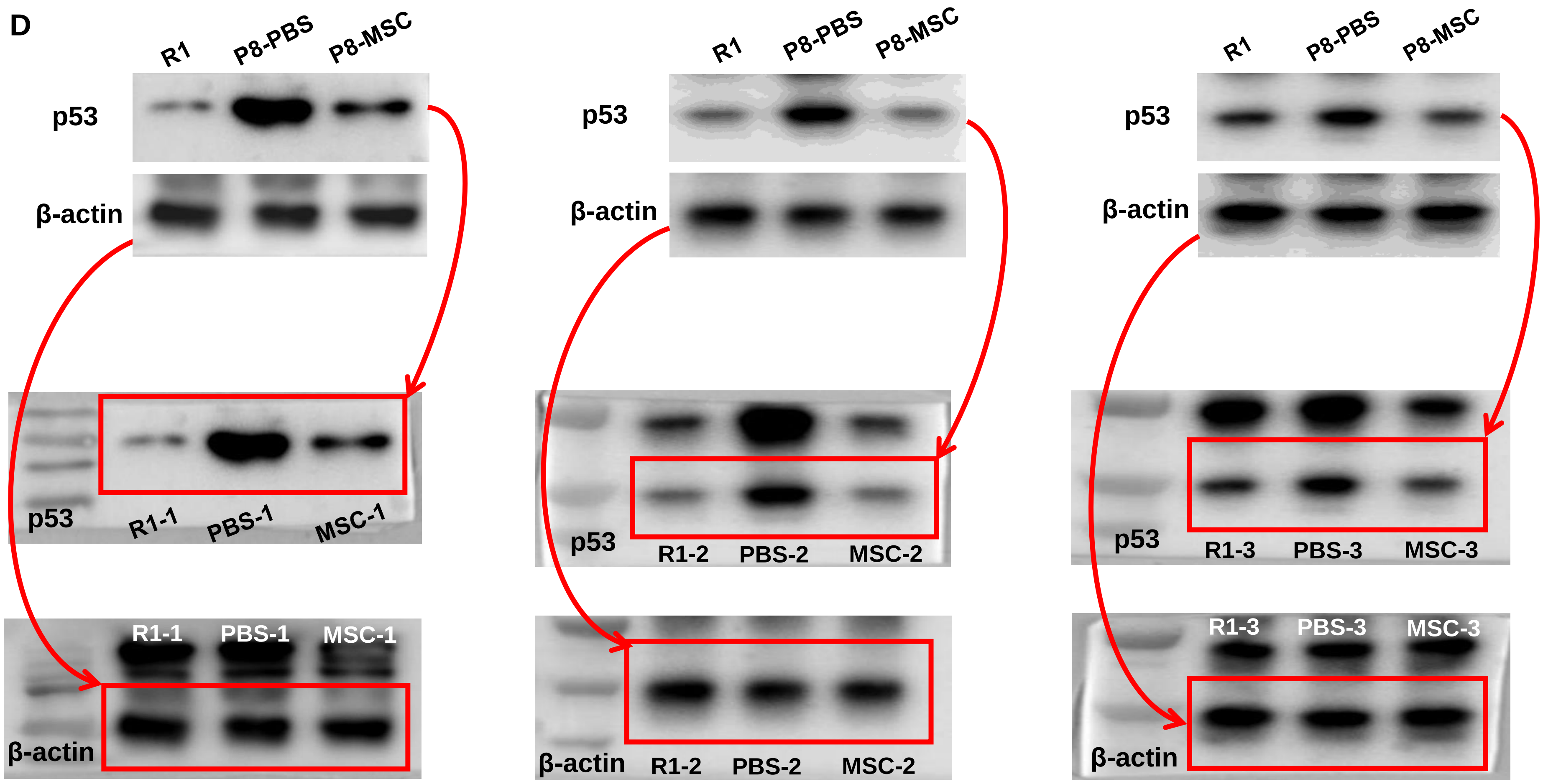

Figure S6. hUC-MSCs increased autophagy and delayed myocyte senescence in D-gal-induced ageing model through p16-Rb / p53-p21 axis.

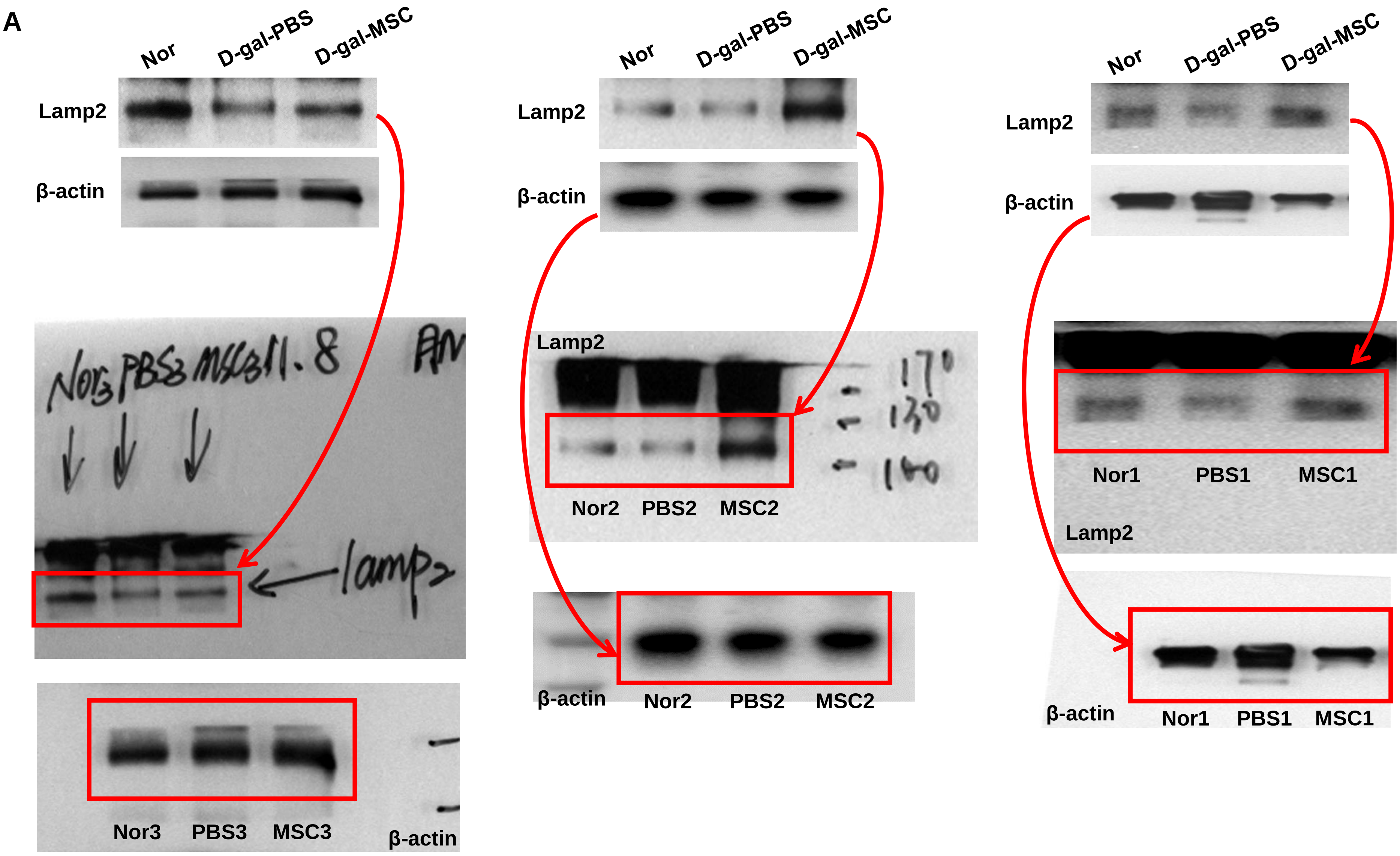

Figure S6. hUC-MSCs increased autophagy and delayed myocyte senescence in D-gal-induced ageing model through p16-Rb / p53-p21 axis.

B

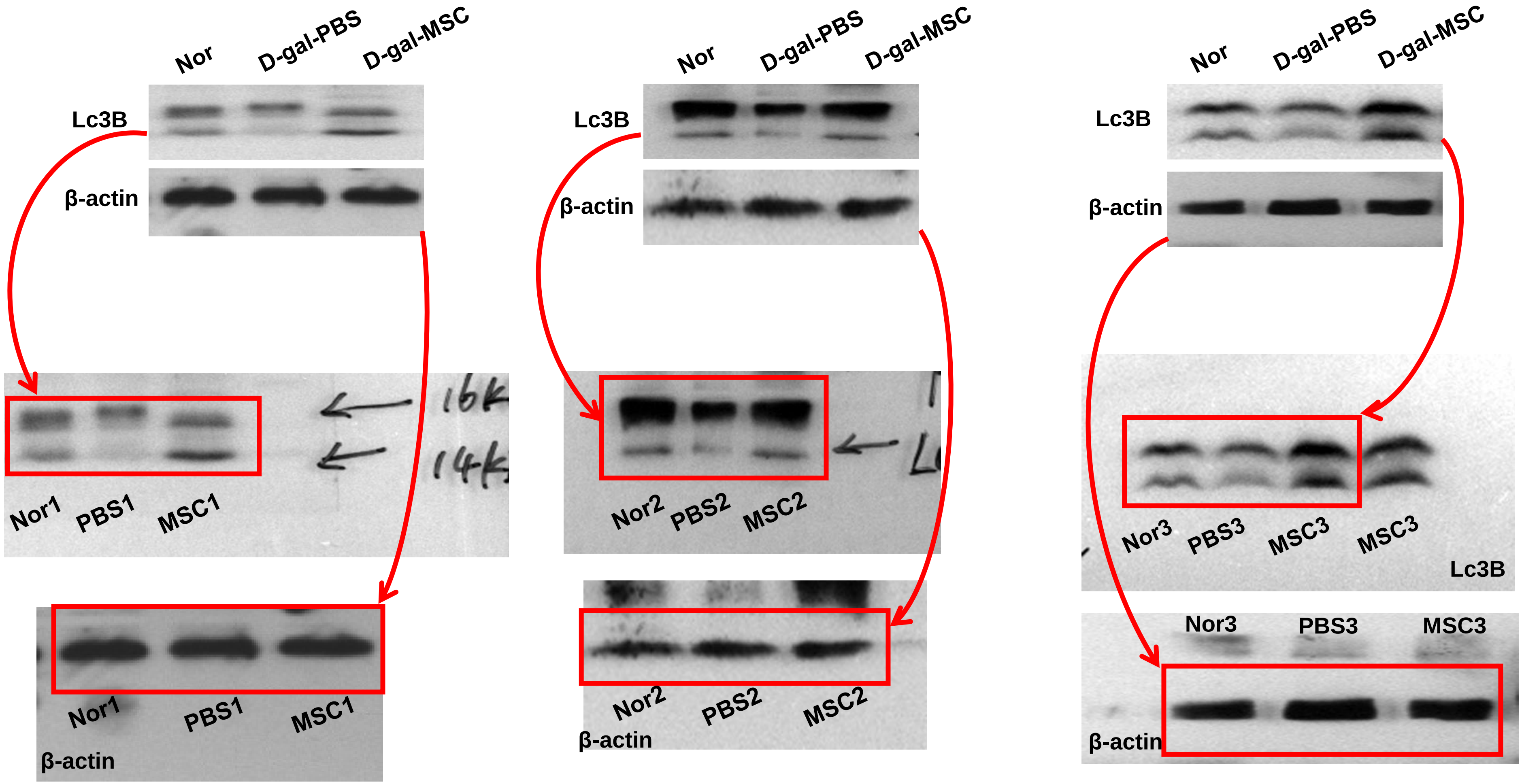

Figure S6. hUC-MSCs increased autophagy and delayed myocyte senescence in D-gal-induced ageing model through p16-Rb / p53-p21 axis.

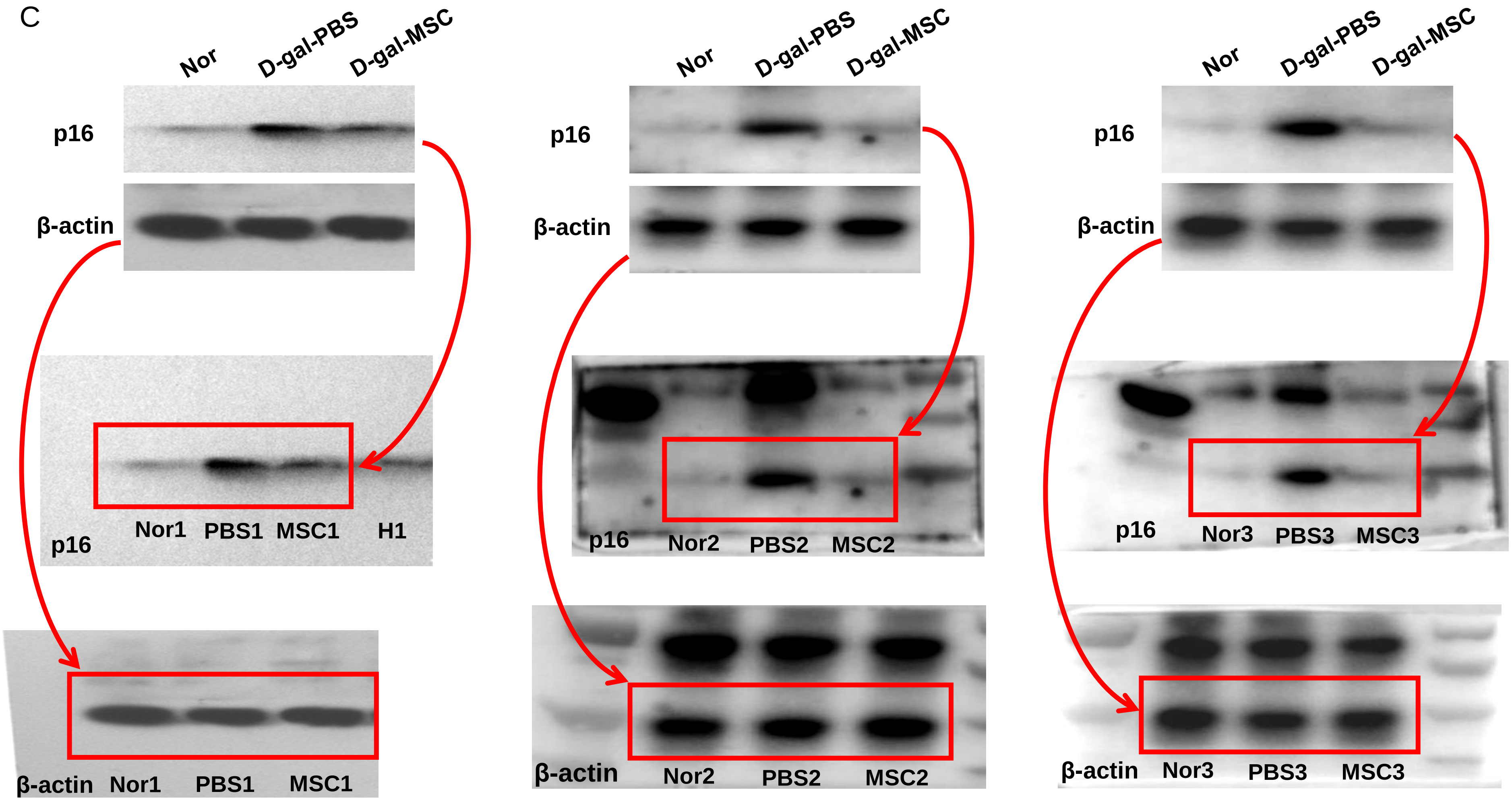

Figure S6. hUC-MSCs increased autophagy and delayed myocyte senescence in D-gal-induced ageing model through p16-Rb / p53-p21 axis.

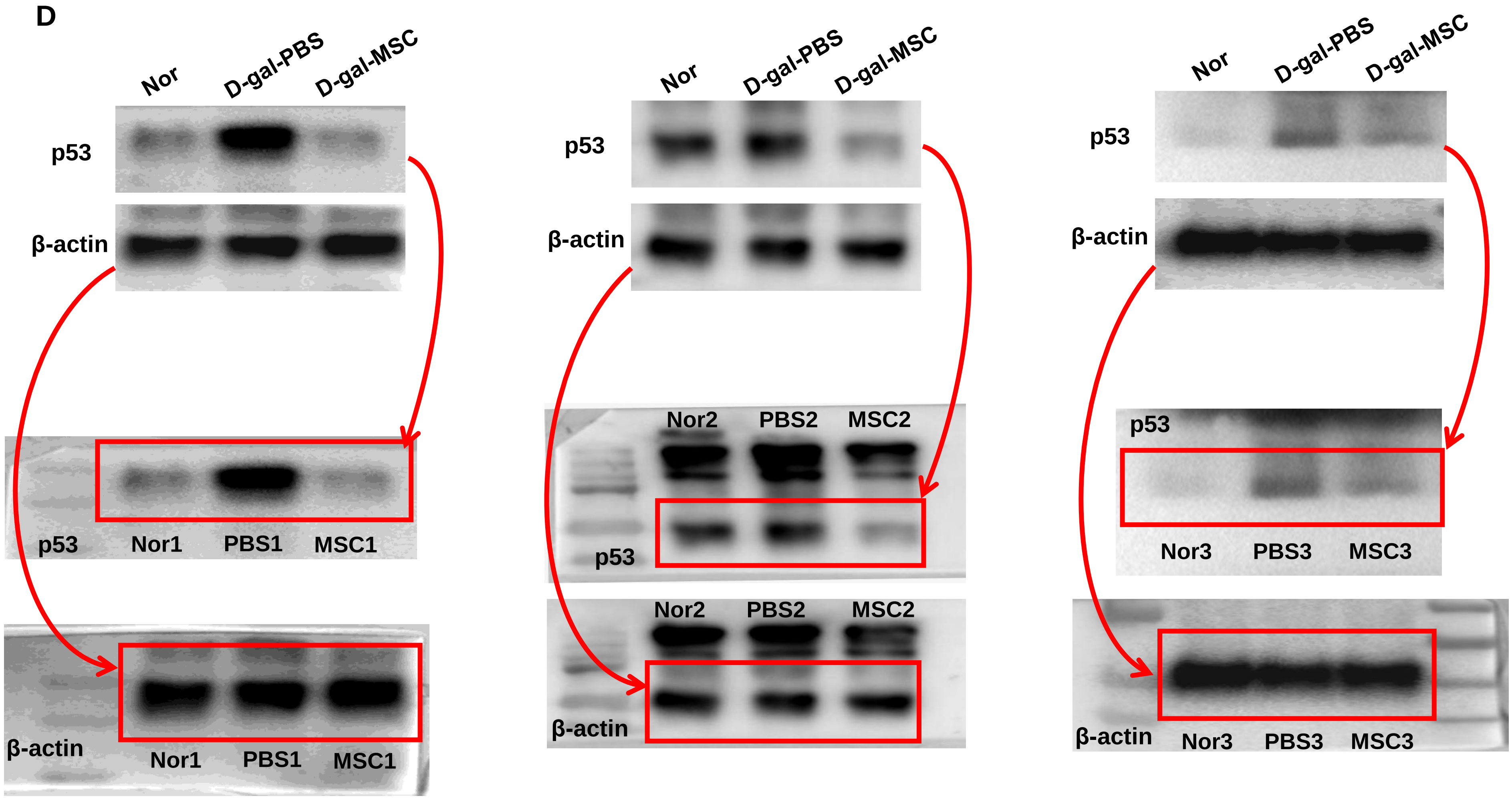

Supplement: Supplementary file 8 — CDDIS-22-3682RR. Original Western Blots [file 41419_2023_5843_MOESM8_ESM.pdf]
